# A unique Cretaceous–Paleogene lineage of piranha-jawed pycnodont fishes

**DOI:** 10.1038/s41598-017-06792-x

**Published:** 2017-07-28

**Authors:** Romain Vullo, Lionel Cavin, Bouziane Khalloufi, Mbarek Amaghzaz, Nathalie Bardet, Nour-Eddine Jalil, Essaid Jourani, Fatima Khaldoune, Emmanuel Gheerbrant

**Affiliations:** 1 Géosciences Rennes, UMR CNRS 6118, Université de Rennes 1, 35042 Rennes, France; 2 Département de Géologie et Paléontologie, Muséum d’Histoire Naturelle de Genève, CP 6434, 1211 Geneva 6, Switzerland; 3 ISYEB – Institut de Systématique, Evolution, Biodiversité, UMR 7205 Muséum National d’Histoire Naturelle, CNRS, EPHE, UPMC, Sorbonne Universités. MNHN, CP38, 75005 Paris, France; 4 OCP Group SA, Avenue Hassan II, BP 168, 25010 Khouribga, Morocco; 5 CR2P – Centre de Recherche sur la Paléobiodiversité et les Paléoenvironnements, UMR 7207, Muséum National d’Histoire Naturelle, CNRS, UPMC, Sorbonne Universités. MNHN, CP38, 75005 Paris, France

**Keywords:** Palaeontology, Taxonomy

## Abstract

The extinct group of the Pycnodontiformes is one of the most characteristic components of the Mesozoic and early Cenozoic fish faunas. These ray-finned fishes, which underwent an explosive morphological diversification during the Late Cretaceous, are generally regarded as typical shell-crushers. Here we report unusual cutting-type dentitions from the Paleogene of Morocco which are assigned to a new genus of highly specialized pycnodont fish. This peculiar taxon represents the last member of a new, previously undetected 40-million-year lineage (Serrasalmimidae fam. nov., including two other new genera and *Polygyrodus* White, 1927) ranging back to the early Late Cretaceous and leading to exclusively carnivorous predatory forms, unique and unexpected among pycnodonts. Our discovery indicates that latest Cretaceous–earliest Paleogene pycnodonts occupied more diverse trophic niches than previously thought, taking advantage of the apparition of new prey types in the changing marine ecosystems of this time interval. The evolutionary sequence of trophic specialization characterizing this new group of pycnodontiforms is strikingly similar to that observed within serrasalmid characiforms, from seed- and fruit-eating pacus to flesh-eating piranhas.

## Introduction

Pycnodonts have been known and studied for more than two centuries^1^ since they are one of the most remarkable fishes present in Konservat-Lagerstätten of Mesozoic and early Cenozoic age (e.g., Solnhofen and Monte Bolca)^2,3^. This group of deep-bodied actinopterygians appeared in the Late Triassic and became extinct during the Eocene epoch, with a peak of diversity occurring during the early Late Cretaceous^2–4^. Their decline started during the latest Cretaceous, and all Cenozoic pycnodonts reported so far belong to a few closely related, taxonomically and ecomorphologically poorly diverse lineages of Pycnodontidae^3,4^. A recent cladistic analysis^5^ has shown that the Pycnodontiformes are not the sister group to the Teleostei as commonly proposed, but rather have a basal position among the Neopterygii. Pycnodontiforms were widely distributed and inhabited pelagic and coastal marine waters as well as brackish and freshwater environments^2,3,6^. They played a significant ecological role in this wide range of aquatic ecosystems^2,3^. The powerful jaws, crushing dentitions and preserved gut contents of pycnodonts clearly indicate predominantly durophagous feeding habits^2,3,7,8^. However, it has recently been argued that some of these fishes were also herbivorous forms able to graze and browse on macroalgae^2,8,9^.

In this study, we report the discovery of peculiar isolated jaw elements (i.e., vomers and prearticulars) from the Paleogene and latest Cretaceous phosphate deposits of the Ouled Abdoun Basin, Morocco. These specimens, characterized by a derived cutting-type dentition, are shown to belong to an unexpected new lineage of macropredatory, flesh-eating pycnodonts. These highly specialized forms represent two new taxa and are interpreted as the last members of a new family that also includes the enigmatic genus *Polygyrodus*^10,11^ and a third new taxon, from the Late Cretaceous of Europe and Niger, respectively. During the evolutionary history of this group of pycnodontiforms, the most remarkable adaptation is the strong reduction of the number of tooth rows in both vomerine and prearticular dentitions combined with the development of sharp labiolingually compressed teeth, documenting a gradual transformation from typical crushing tooth plates towards slicing blades. Our findings indicate that the last pycnodontiforms were represented during the Paleogene by two main groups which appear disparate, both ecologically and phylogenetically. Interestingly, this new group of pycnodonts shows convergent evolution with serrasalmid fishes, a modern group including the durophagous pacus and the famous piranhas^12,13^.

## Results

**Systematic Palaeontology**

Osteichthyes Huxley, 1880

Actinopterygii Cope, 1887

Pycnodontiformes Berg, 1937

**Serrasalmimidae** fam. nov.

**Included genera**. *Serrasalmimus* gen. nov. (type genus), *Eoserrasalmimus* gen. nov., *Damergouia* gen. nov., *Polygyrodus* White, 1927.

**Diagnosis**. Medium-sized to large-sized (up to 1 m) pycnodontiform fishes only known by isolated vomerine and prearticular dentitions and distinguished by the following unique combination of characters: presence of monocuspid or bicuspid mammiform teeth with cingulum, due to the presence of a modified central papilla; most or all teeth of main rows elevated, longer and higher than wide; prearticular symphysis short anteroposteriorly, about half or less than half the bone length.

**Remark**. The suite of characters that allows to refer the Serrasalmimidae to the Pycnodontiformes is described below (discussion part) and in Supplementary Text S1, part H.

***Serrasalmimus secans*** gen. et sp. nov.

**Figure 1:**
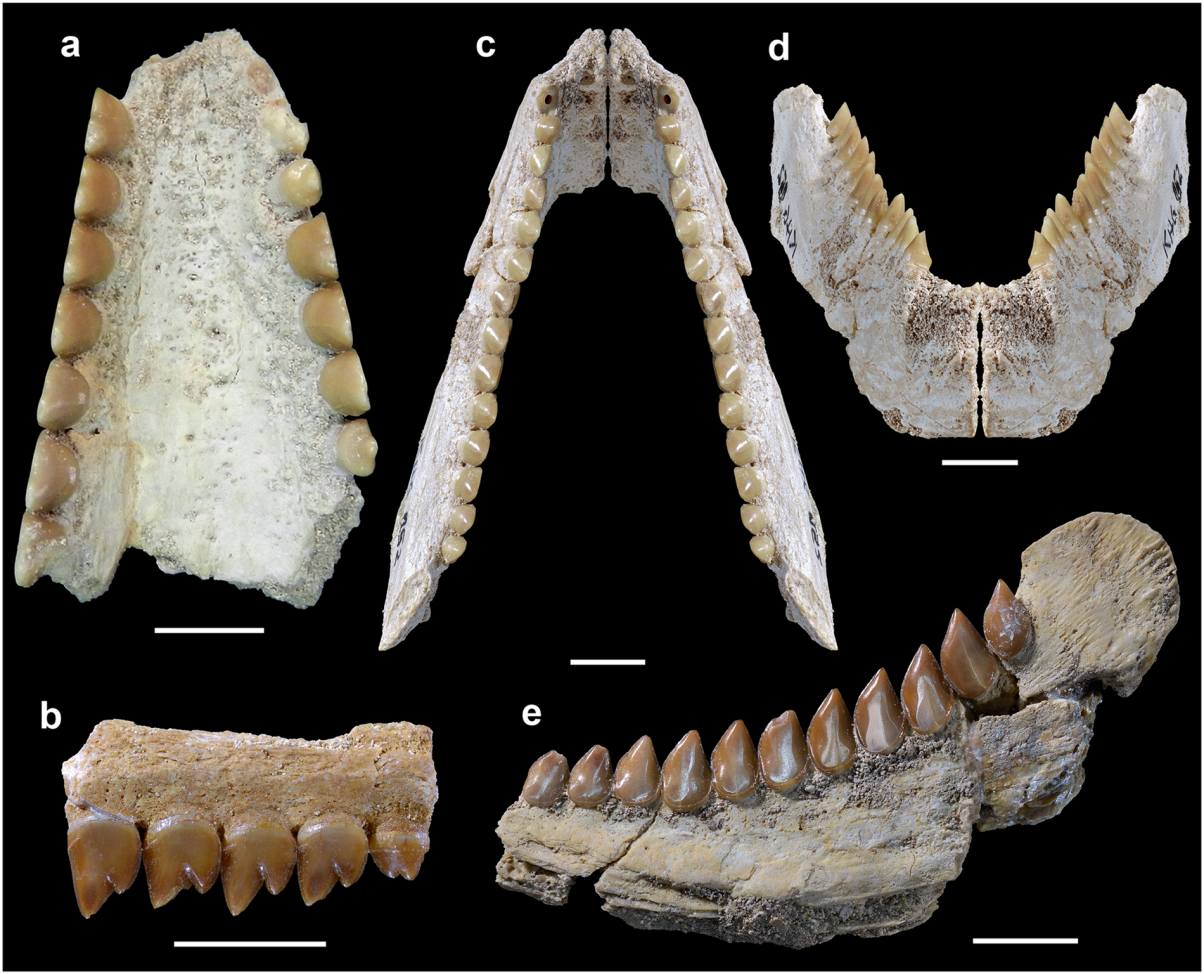
Jaw elements and dentition of *Serrasalmimus secans* gen. et sp. nov. from the Paleogene of Morocco. **(a)** OCP DEK-GE 701, vomer in ventral (occlusal) view. (**b)** MHNM KHG 163, fragmentary vomer in right lateral (labial) view. (**c**,**d)** MHNM KHG 152, left prearticular (and mirror image for the right side) in dorsal (occlusal) (**c**) and anterior view (**d**). (**e)** MHNM KHG 158, right prearticular in medial (lingual) view. Scale bars, 10 mm. (**b**–**e**, photographs by Lilian Cazes – CNRS/MNHN).

**Figure 2:**
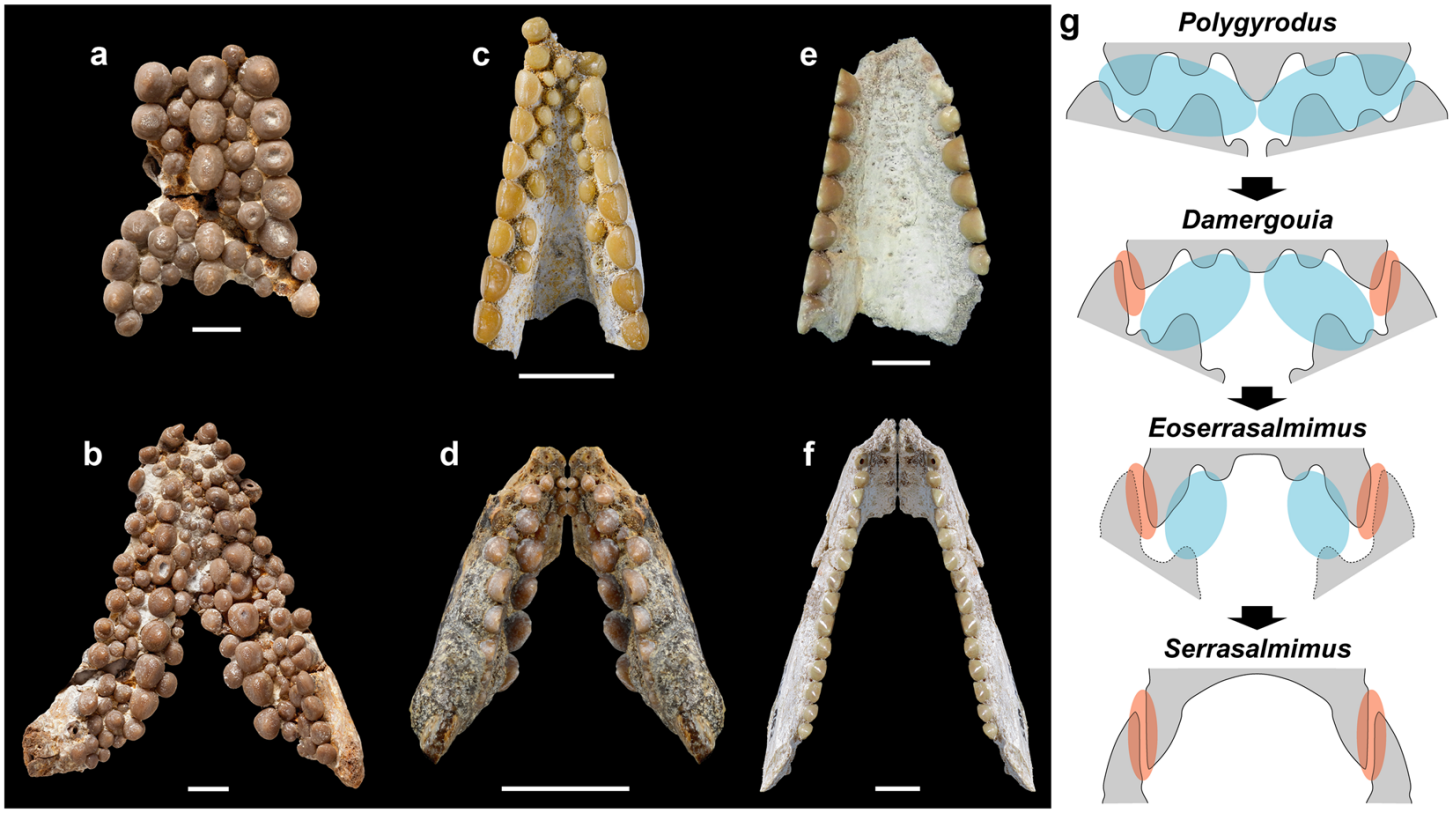
Jaw elements and dentitions of serrasalmimid fishes, and evolutionary shift from crushing to cutting function. **(a**,**b)** *Polygyrodus cretaceus* (Agassiz, 1843) from the Turonian of England, NHMUK PV OR 39048, vomer (**a**) and NHMUK PV P 11157, right and left prearticulars (and dentaries?) (**b**) in ventral and dorsal views, respectively. (**c)** *Eoserrasalmimus cattoi* gen. et sp. nov. from the Maastrichtian of Morocco, MHNM KHG 165, vomer in ventral view. (**d)** *Damergouia lamberti* gen. et sp. nov. from the Turonian of Niger, MNHN.F.HGS176, left prearticular (and mirror image for the right side) in dorsal view. (**e**,**f)** *Serrasalmimus secans* gen. et sp. nov., OCP DEK-GE 701, vomer (**e**) and MHNM KHG 152, left prearticular (**f**) in ventral and dorsal views, respectively. (**g)** Schematic diagram showing the evolutionary sequence of morphological and functional changes observed in serrasalmimid jaws. The jaws are shown in cross section and with teeth in occlusion; the morphology of the teeth has been simplified, and the shape of the unknown lower dentition of *Eoserrasalmimus* has been inferred from that of the upper dentition. Blue and red shadings indicate crushing and cutting surfaces, respectively. Note the rotation of the toothed surface of the prearticulars, from a nearly horizontal to a vertical position. Scale bars, 10 mm; sketches in (**g**) not to scale. (**a**,**b**, copyright: The Trustees of the Natural History Museum, London – http://data.nhm.ac.uk; **c**,**d**,**f**, photographs by Lilian Cazes – CNRS/MNHN).

**Holotype**. OCP DEK-GE 701, a nearly complete vomer (Figs 1a and 2e and S1a–d).

**Etymology**. The generic name is a combination of *Serrasalmus* and *μίμος* (Greek), *mimus* (Latin spelling), meaning mimic, in reference to the resemblance between the dentition of the new taxon and that of piranhas. The specific epithet is from *secare* (Latin), meaning cutting, carving up, in reference to the function of the jaws of the new taxon.

**Referred material**. See Supplementary Text S1, part A for details (Figs 1b–e and S1e–k and S2).

**Type locality and horizon**. Eastern Ouled Abdoun Basin, Province of Khouribga, Morocco. “Big coprolite” Bone Bed, Upper Phosphorite Bed IIa, early Thanetian (Paleocene) in age^14^.

**Diagnosis**. Large-sized serrasalmimid pycnodontiform fish characterized by a cutting-type dentition and distinguished by the following autapomorphies: vomerine dentition consisting of two rows (persistence of only the two lateralmost rows) of slightly overlapping teeth; small mammiform teeth restricted to the anteriormost portion of the vomerine and prearticular dentitions; vomerine rows with numerous (at least up to nine) labiolingually compressed, elevated bicuspid teeth with both apices slightly bent posteriorly; posterior cusp of vomerine bicuspid teeth more developed than anterior cusp; anterior cusp of vomerine bicuspid teeth decreasing in size posteriorly and tending to disappear in posteriormost teeth; vomerine teeth showing a linguobasal cingulum and a large vertical wear facet developed on most of the labial face; edentulous medial area of the ventral surface of the vomer concave in anteroposterior (cross-sectional) view; strongly flattened, labiolingually compressed prearticular bone; prearticular dentition consisting of one row (persistence of only the lateralmost row) of slightly overlapping teeth; prearticular row with numerous (up to fifteen) labiolingually compressed, triangular, elevated monocuspid teeth with an apex slightly bent posteriorly; prearticular teeth showing a linguobasal cingulum and a large wear facet developed on most of the lingual face; prearticular tooth row curved (concave dorsally) in lateral view; prearticular symphysis reduced in length, restricted to the anterior part of the bone (about one-quarter the prearticular length); symphysial area suboval to subtrapezoidal in shape, oblique in medial view.

**Description**. See Supplementary Text S1, part A.

***Eoserrasalmimus cattoi*** gen. et sp. nov.

**Holotype**. MHNM KHG 165, a complete vomer (Figs 2c and S3a–d).

**Etymology**. The generic name is a combination of *ἕως* (Greek), *eos* (Latin spelling), meaning dawn, and *Serrasalmimus*, in reference to the early (pre-K/Pg boundary) occurrence of the new taxon closely related to the Paleogene genus *Serrasalmimus*. The specific epithet refers to Mr Patrick Catto, who kindly donated the specimen described here.

**Type locality and horizon**. Eastern Ouled Abdoun Basin, Province of Khouribga, Morocco. Phosphorite Bed III, late Maastrichtian (Late Cretaceous) in age^14^.

**Diagnosis**. Medium-sized serrasalmimid pycnodontiform fish characterized by a crushing/cutting-type dentition and distinguished by the following autapomorphies: vomerine dentition consisting of two main tooth rows (i.e., the two lateralmost rows), each flanked medially by a secondary, shorter row of reduced teeth; main rows with numerous (up to nine) slightly labiolingually compressed, relatively low bicuspid teeth; edentulous medial area of the ventral surface of the vomer depressed in its posterior portion.

**Description**. See Supplementary Text S1, part B.

***Damergouia lamberti*** gen. et sp. nov.

**Holotype**. MNHN.F.HGS176 (ex-Lambert’s collection), a left prearticular with nearly complete dentition (Figs 2d and S3e–h).

**Etymology**. The generic name is from the geographical origin (Damergou, Niger) of the new taxon. The specific epithet refers to Mr Roger Lambert, who collected the two specimens described here.

**Referred material**. MNHN.F.HGS177 (ex-Lambert’s collection), a fragmentary vomer (Supplementary Fig. S3i–k).

**Type locality and horizon**. “Locality n° AD 074 of the sampling zone III”, 4 km SSE of Tanout, Damergou area, Niger^15^. “Lower level (n° 2) of section 2”, Turonian (Late Cretaceous) in age^15,16^.

**Diagnosis**. Medium-sized serrasalmimid pycnodontiform fish characterized by a crushing/cutting-type dentition and distinguished by the following autapomorphies: prearticular dentition consisting of two main rows of labiolingually compressed, triangular, elevated monocuspid teeth; lateral main row consisting of five teeth with a well-developed lingual wear facet and an apex slightly bent posteriorly; medial main row consisting of four teeth with an apex slightly bent anteriorly; presence on the medial face of the prearticular of a well-developed, flattened, triangular edentulous shelf between the toothed area and the ventromedial margin; symphysis area subtrapezoidal in shape, oblique in medial view.

**Description**. See Supplementary Text S1, part C.

## Discussion

### Evolutionary history of serrasalmimid pycnodonts

Although very derived, the three new taxa (i.e. *Serrasalmimus*, *Eoserrasalmimus*, and *Damergouia*) can be referred to the Pycnodontiformes with confidence (see Supplementary Text S1, part H). They are excluded from any non-actinopterygian clades, in particular the squamates with a roughly similar tooth morphology and dentition (e.g., agamid lizards), by the histological structure of the teeth, by the mode of occlusion, and by osteological characters of the tooth-bearing bones. The jaw elements and dentitions of *Eoserrasalmimus* and *Serrasalmimus* show superficial resemblance to those of some other actinopterygians, especially teleosts (e.g., characiforms, pachycormiforms, saurodontids), but osteological features readily contradict such interpretations. Contrariwise, all genera described here (including the highly specialized genus *Serrasalmimus*) show a combination of jaw characters that is fully compatible with an assignment to the Pycnodontiformes (e.g., vomer single and median, median dorsal crest of the vomer, prearticular symphysis strong and rugose, stout coronoid process arising from the posterolateral side of each prearticular, vomer fitting into the mandible and upper dentition occluding lingually to lower dentition, woven pattern of acrodin bundles in the outer tooth layer, thin and straight tubules penetrating from the dentine into the acrodin, single-layered acrodin comprising only one type of tubules)^8^, including several synapomorphies of this group (see Supplementary Text S1, part H for details and references). This combination of traits makes the three new taxa unambiguous pycnodontiforms.

*Polygyrodus*, from the Late Cretaceous of Europe (see revised diagnosis and distribution of this genus in Supplementary Text S1, part D), is a peculiar monospecific genus known by isolated crushing-type dentitions bearing unique serrasalmimid characters, such as the presence of elevated and longer than wide mammiform teeth with cingulum^10,11^ (Figs 2a,b and S3n–q). The latter genus and the three genera described above—*Polygyrodus*, *Damergouia*, *Eoserrasalmimus* and *Serrasalmimus*—exemplify an evolutionary sequence from a chiefly crushing dentition typical of pycnodont fishes towards a highly specialized cutting dentition without superficial resemblance with other members of this clade.

We ran a cladistic analysis (see Supplementary Text S1, part E, and Supplementary Table S2) to determine the phylogenetic relationships of the four serrasalmimid genera to other pycnodontiforms, keeping in mind that dental characters of pycnodont fishes contain a poor phylogenetic signal^17^. The highly autapomorphic features of the studied specimens required the definition of new characters that were used in the analysis (see Supplementary Text S1, part E). In the most extensive cladistic analysis of the Pycnodontiformes carried out to date^18^, *Gyrodus* has a single autapomorphic dental character (i.e., the presence of a well-marked central papilla on vomerine and prearticular teeth), which is clearly shared with *Polygyrodus*. In the latter genus, this central papilla becomes higher and slightly compressed labiolingually. In the most derived predatory serrasalmimids with a cutting dentition, this central papilla is strongly modified to form the single or bifid sharp cusp.

**Figure 3:**
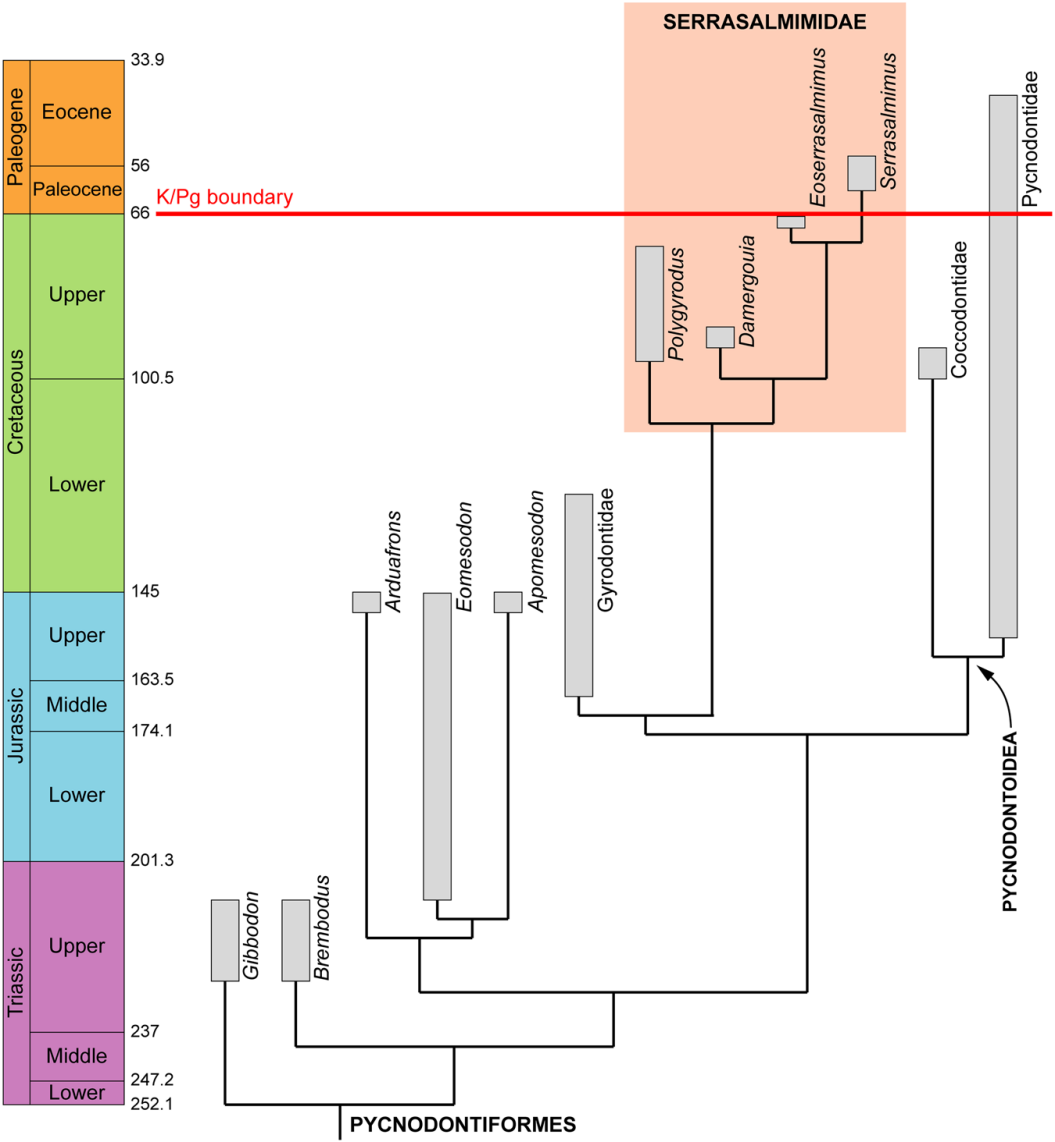
Calibrated phylogenetic hypothesis of pycnodontiform interrelationships. This cladogram (based on a simplified version of the tree shown in Supplementary Fig. S4) shows the disparate origins of Paleogene pycnodont fishes, which are represented by the serrasalmimid *Serrasalmimus* and by a few members of the Pycnodontidae.

The two main results of the phylogenetic analysis are: (1) the monophyly of the Serrasalmimidae is confirmed, and (2) the Serrasalmimidae are found to be the sister group to the Gyrodontidae (Figs 3 and S4). The evolutionary link between these two families could be exemplified by possible stem serrasalmimids such as MB.f.7233, an isolated prearticular dentition from an Early Cretaceous erratic block of the Baltic Sea which was tentatively assigned to aff. *Gyrodus*? sp.^19^. This specimen shows similarities to the prearticular dentition of *Polygyrodus*, such as five rows of subequally sized teeth and globular tooth crowns with a cingulum. In our phylogenetic analysis, *Gyrodus* and the Serrasalmimidae are recovered in a more derived position than *Gibbodon*, *Brembodus*, *Arduafrons*, *Eomesodon* and *Apomesodon* but basal to the taxonomically, morphologically and ecologically more diverse Pycnodontoidea (i.e., the superfamily comprising the families Pycnodontidae and Coccodontidae)^3,20^ (Figs 3 and S4).

Most of the post-Jurassic pycnodonts belong to the Pycnodontoidea, and the Cretaceous radiation of this group is well documented by a rich fossil record^3,21^. Characterized by a drastic decrease in diversity and disparity, the Cenozoic pycnodonts reported so far correspond to a few genera of Pycnodontidae (i.e., *Pycnodus*, *Oropycnodus*, *Nursallia*, *Abdobalistum*, and *Palaeobalistum*)^3,20,21^. Our results indicate that an ancient, but specialized, lineage of non-pycnodontoid pycnodontiforms also survived the end-Cretaceous mass extinction, thus revealing disparate phylogenetic origins for Paleogene pycnodonts (Fig. 3). The discovery of the new family Serrasalmimidae shows that pycnodonts basal to the Pycnodontoidea were ecomorphologically more diverse than previously thought and provides additional evidence of the remarkable plasticity of shape and diet within the Pycnodontiformes. This new family, whose earliest known member is from the Cenomanian–Turonian, represents an additional surprising facet of the explosive radiation of the Pycnodontiformes in early Late Cretaceous.

### Convergence between serrasalmimid and serrasalmid fishes

**Figure 4:**
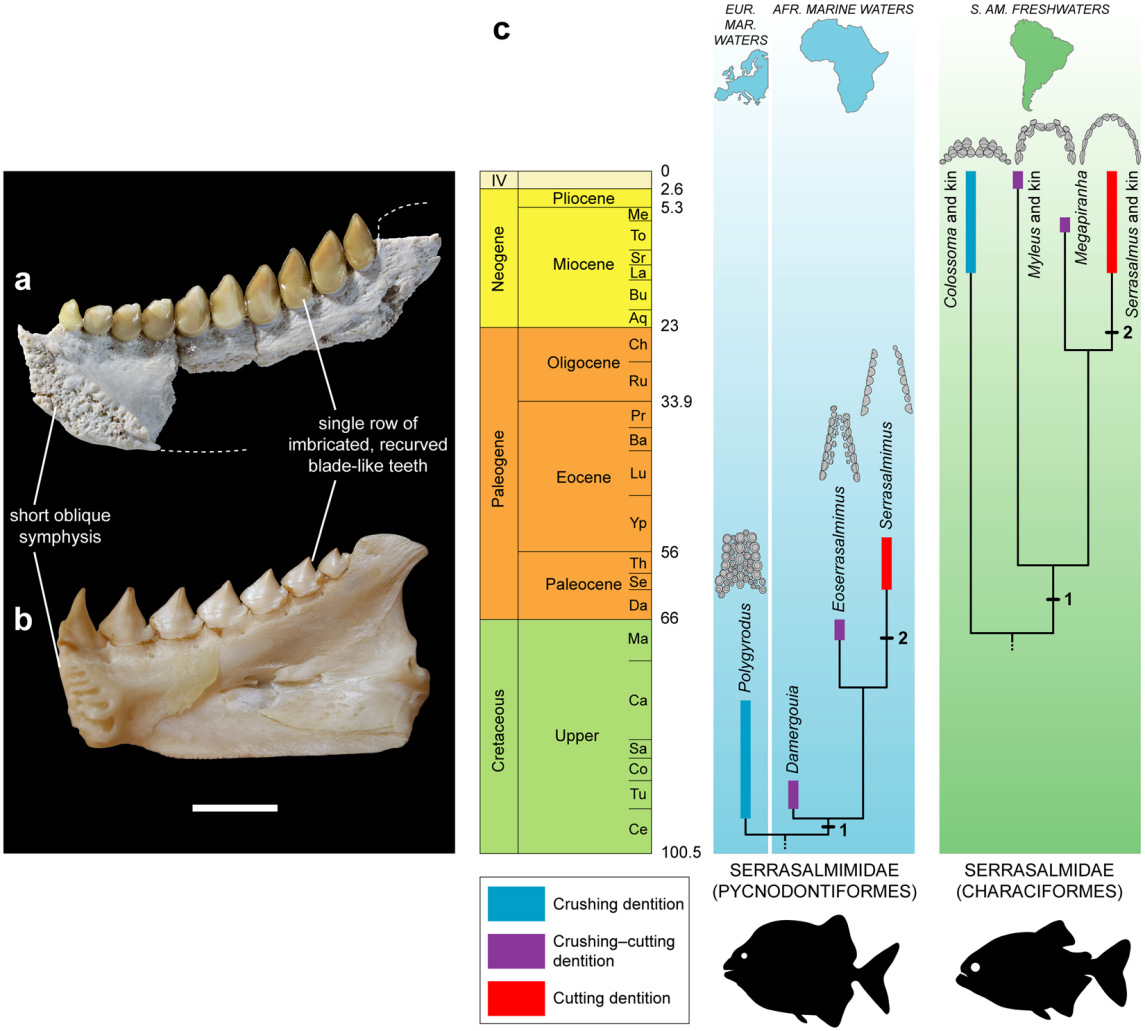
Convergence between serrasalmimid and serrasalmid fishes. (**a**,**b)** Comparison between a right prearticular (MHNM KHG 159) of *Serrasalmimus secans* gen. et sp. nov. (**a**) and a right dentary of extant *Serrasalmus rhombeus* (**b**) from French Guyana (unnumbered specimen from P.-Y. Lebail’s collection, Fish Physiology and Genomics Institute, INRA LGPG, Rennes), in medial view (note that the articular and the angular are still in articulation in the posterior part of the mandible). (**c)** Phylogenetic relationships between the four genera of serrasalmimid fishes (Pycnodontiformes) (supported by our phylogenetic analysis; see Figs 3 and S4), and convergent evolution with serrasalmid fishes (Characiformes). A congruent series of transformations in dental morphology and arrangement is observed in serrasalmimids and serrasalmids. In both groups, the transition from crushing to cutting dentitions (upper jaws are represented here, with vomerine dentition for serrasalmimids and premaxillary dentition for serrasalmids) is characterized by the successive acquisition of two key features: labiolingually compressed teeth (character 1) and single row of blade-like teeth (character 2). See Supplementary Fig. S7 for further information about the serrasalmid dentitions represented here. Stratigraphic distribution and time-calibrated phylogeny of the serrasalmid genera from refs^22,28^. Stage abbreviations are as follows: Aq, Aquitanian; Ba, Bartonian; Bu, Burdigalian; Ca, Campanian; Ce, Cenomanian; Ch, Chattian; Co, Coniacian; Da, Danian; La, Langhian; Lu, Lutetian; Ma, Maastrichtian; Me, Messinian; Pr, Priabonian; Ru, Rupelian; Sa, Santonian; Se, Selandian; Sr, Serravalian; Th, Thanetian; To, Tortonian; Tu, Turonian; Yp, Ypresian. Scale bar, 10 mm. (**a**, photograph by Lilian Cazes – CNRS/MNHN).

The jaw and tooth morphologies of serrasalmimids reveal a novel, unexpected trophic specialization for pycnodont fishes, from durophagous/omnivorous forms (i.e., *Polygyrodus*) to predatory, flesh-eating forms (i.e., *Serrasalmimus*) (Fig. 2; see Supplementary Text S1, part F). This evolutionary sequence (Fig. 2g) is strikingly similar to that observed in the dentitions of the Serrasalmidae (Characiformes), i.e. between the pacu clade, the *Myleus* clade, the extinct genus *Megapiranha*, and the piranha clade^12,13,22^. In both cases, a decrease in tooth row number and a labiolingual compression of the teeth occur, leading to the acquisition of a single row of imbricated teeth in each jaw^13,23^ (Figs 4 and S7; see Supplementary Text S1, parts F, G). In addition, the acrodin layer of *Serrasalmimus* teeth is similar in thickness to that of *Serrasalmus* teeth (see Supplementary Fig. S5 and ref.^24^). However, four main anatomical differences can be noted between the jaws and teeth of the two families. In serrasalmids, this evolutionary sequence affects the premaxillary and dentary dentitions^23^, whereas it concerns the vomerine and prearticular dentitions in serrasalmimids. Second, as a consequence, the upper dentition is labial to the lower dentition in serrasalmids^23^, conversely to the condition observed in serrasalmimids and more generally in pycnodonts. Third, serrasalmids have an acrodont tooth implantation with a direct fibrous attachment^23,24^, whereas an ankylothecodont-like condition is present in serrasalmimids, characterized by the absence of true sockets and by teeth showing a tubular root-like structure firmly fused to the bone (as in other pycnodonts; see ref.^8^, Figs 23, 45) (Supplementary Fig. S1k). Fourth, the superficial acrodin layer of serrasalmid teeth shows longitudinally oriented sheets of crystals^24^, whereas strongly woven fibre bundles are present on the crown surface of pycnodont teeth (Supplementary Fig. 5g).

### Feeding behaviour of *Serrasalmimus*

**Figure 5:**
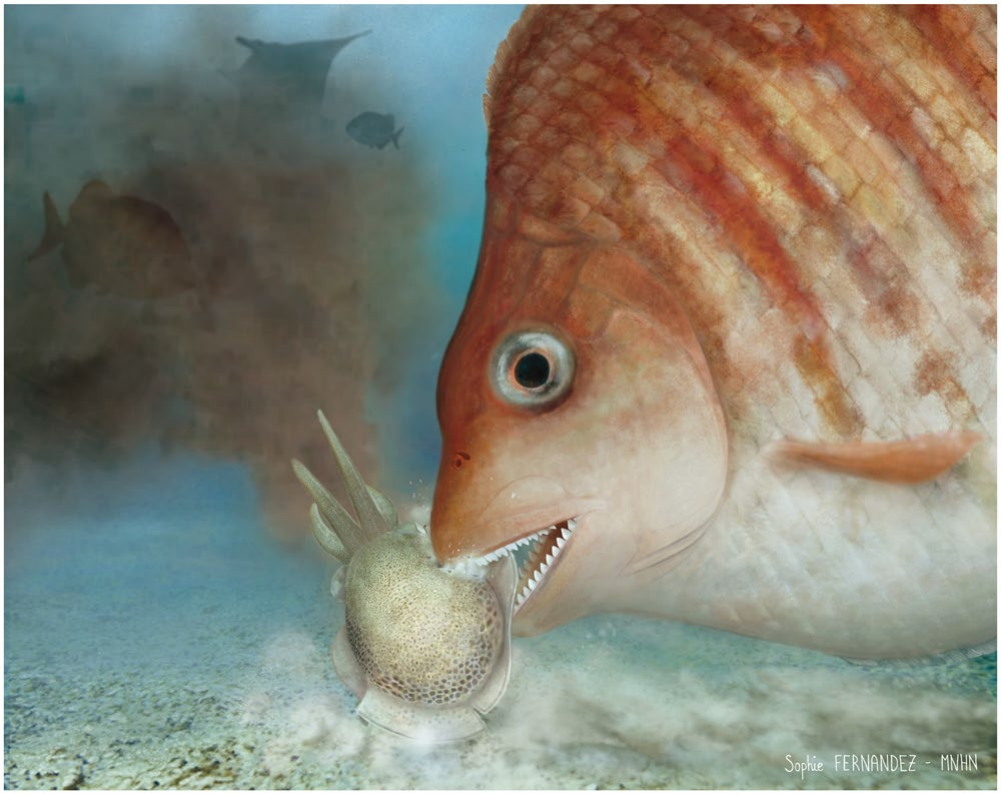
Life reconstruction of *Serrasalmimus secans* gen. et sp. nov. This reconstruction illustrates the putative feeding behaviour of this piranha-jawed pycnodont, as predator of soft-bodied animals such as cuttlefishes (foreground) and facultative ectoparasite of large animals such as primitive devil rays (background). The head, squamation and body outline are based on *Gyrodus*, the most closely related pycnodont taxon known from complete skeletons (Artwork by Sophie Fernandez – MNHN).

The cutting dentition and flesh-eating habits developed within serrasalmimid fishes were possibly linked to the apparition and diversification of a new group of soft-bodied prey items. Interestingly, the Sepiida (cuttlefishes *s*.*l*.) are demersal coleoid cephalopods that seem to have originated and diversified in the western part of the Tethys (i.e., shelf seas of Western Europe and North Africa) during the Late Cretaceous–Paleocene interval^25^. Sepiids might have represented an important food source for the last members of the Serrasalmimidae, characterized by a derived, effective cutting dentition (Fig. 5). These flesh-eating serrasalmimids were probably also able to prey upon small fishes and other shell-less, soft-bodied animals such as jellyfishes. *Serrasalmimus* might also have developed a facultative ectoparasitic foraging strategy consisting of biting fins of large animals, like in some modern fishes with a specialized cutting dentition (i.e., cookiecutter sharks and piranhas; Figs 4b and S7c and S8; see Supplementary Text S1, part G). Butterfly, eagle and devil rays (Gymnuridae, Myliobatidae and Mobulidae, respectively), whale sharks (Rhincodontidae) and sea turtles (Bothremydidae and Cheloniidae)—all well represented in the Paleogene biotas of the Ouled Abdoun Basin^26,27^—might have been targeted by these piranha-jawed pycnodonts.

## Material and Methods

The single *Eoserrasalmimus* specimen from the phosphate series of the Ouled Abdoun Basin, Morocco, was surface-collected from mixed Maastrichtian deposits of the Bed III. The stratigraphic origin of this specimen is confirmed by the color of the fossil and the characteristics of the matrix. The *Serrasalmimus* specimens from the phosphate series of the Ouled Abdoun Basin were collected *in situ* or surface-collected from mixed Paleocene and Eocene deposits coming from the Beds II and I, respectively. The stratigraphic origin of the specimens not excavated *in situ* has been inferred from the color of the fossil and the characteristics of the matrix (see Supplementary Table S1).

The specimens described herein are permanently housed in the collections of the Muséum d’Histoire Naturelle de Marrakech, Morocco (MHNM), the Muséum National d’Histoire Naturelle, Paris, France (MNHN), the Natural History Museum, London, United Kingdom (NHMUK) and the Office Chérifien des Phosphates, Khouribga, Morocco (OCP).

For structural and histological observations, a *Serrasalmimus* tooth was removed from a vomer fragment (specimen MHNM KHG 162) and was sectioned longitudinally. The posterior half of this tooth was etched a few minutes with HCl and subsequently observed under scanning electron microscope (SEM).

This published work and the nomenclatural acts it contains have been registered in ZooBank, the proposed registration system for the International Code of Zoological Nomenclature (ICZN). The ZooBank LSIDs (Life Science Identifiers) can be resolved and the associated information viewed through any standard web browser by appending the LSID to the prefix ‘http://zoobank.org/’. The LSID for this publication is: urn:lsid:zoobank.org:pub:4854B81F-2261–41C5-ADE1-6BA2431B644E; the LSID for Serrasalmimidae is: urn:lsid:zoobank.org:act:53EAFE43-63BC-4991-810A-DB8594823453; The LSID for *Serrasalmimus* is: urn:lsid:zoobank.org:act:C5A5C72A-AD6C-49B9-BFF6-2C87DC09E61B; The LSID for *Serrasalmimus secans* is: urn:lsid:zoobank.org:act:BB6050A7-2658-431D-BD47-28688CE9D1B3; The LSID for *Eoserrasalmimus* is: urn:lsid:zoobank.org:act:EE042616-812B-4A06-B36C-3ED85DC0259E; The LSID for *Eoserrasalmimus cattoi* is: urn:lsid:zoobank.org:act:4F1D372B-8FC6-45A5-AE29-16AA1CB41C66; The LSID for *Damergouia* is: urn:lsid:zoobank.org:act:9CD4125F-57ED-4E5F-9BC0-7F197F63C8DC; The LSID for *Damergouia lamberti* is: urn:lsid:zoobank.org:act:E0D62E9B-8141-42C3-8C41-79553C2547C8.

## Electronic supplementary material

Supplementary Information
