# Supplementary material for: A unique Cretaceous–Paleogene lineage of piranha-jawed pycnodont fishes
Source: Sci Rep. 2017 Jul 28;7:6802. doi: 10.1038/s41598-017-06792-x (PMC5533729; doi:10.1038/s41598-017-06792-x)
Supplement: Supplementary file 1 — Supplementary Information [file 41598_2017_6792_MOESM1_ESM.pdf]

SUPPLEMENTARY INFORMATION

A unique Cretaceous–Paleogene lineage of piranha-jawed pycnodont fishes

Romain Vullo, Lionel Cavin, Bouziane Khalloufi, Mbarek Amaghzaz, Nathalie Bardet, Nour-Eddine Jalil, Essaid Jourani, Fatima Khaldoune, Emmanuel Gheerbrant

Table of contents:

Supplementary Text S1

Part A. Description and distribution of *Serrasalmimus secans* gen. et sp. nov.

Part B. Description and distribution of *Eoserrasalmimus cattoi* gen. et sp. nov.

Part C. Description and distribution of *Damergouia lamberti* gen. et sp. nov.

Part D. Revised diagnosis and distribution of *Polygyrodus cretaceus* (Agassiz, 1843)

Part E. Phylogenetic analysis

Part F. Carnivorous specialization of the Serrasalmimidae

Part G. Analogous trophic specialization in other fish groups

Part H. Comments on the taxonomic assignment of *Serrasalmimus*

Part I. Supplementary references

Supplementary Tables S1–S2

Supplementary Figures S1–S9

Supplementary Text S1

Part A. Description and distribution of *Serrasalmimus secans* gen. et sp. nov.

Description of the specimens studied

OCP DEK-GE 701 (holotype; Figs 1a, 2e and Supplementary Fig. S1a–d) is a nearly complete vomer (49 mm in preserved length). Seven and five teeth are preserved in the right and left row, respectively. The anterior portion of the bone show several additional teeth that are broken at the crown/root boundary. In anterior view, the edentulous ventral surface is markedly concave (Supplementary Fig. S1d). A slight anteroposterior ridge is present on each lateral surface of the bone, parallel to and a few millimeters above the tooth row. The dorsal surface shows some large foramina and bears the base of the median dorsal crest of the vomer. This crest does not extend up to the anterior portion of the bone.

MHNM KHG 164a–c represents three toothed fragments that likely belong to the same vomer. The largest fragment (MHNM KHG 164a; Supplementary Fig. S1g, h) is 49 mm in preserved length. Eight teeth are preserved on each row. The largest teeth are 9 mm high. An anteroposterior ridge is present on the lateral surface of the bone. The anteriormost portion of the dentition and the medial region are not preserved.

MHNM KHG 163 (Fig 1b and Supplementary Fig. S1i, j) is a fragmentary vomer (25 mm in preserved length) with five teeth of the anterior portion of the right row preserved. The anteriormost tooth corresponds to the reduced, subconical tooth morphotype.

MHNM KHG 162 (Supplementary Fig. S1k) is a fragmentary vomer (32 mm in preserved length) with four teeth of the posteriormost portion of the right row preserved. This specimen is still included in matrix and seen in lingual view. The teeth are deeply rooted (presence of a single root-like structure as high as the crown) into the jaw and firmly fused to the bone (ankylothecodont-like tooth attachment). A longitudinal (labiolingual) section of anteriormost tooth of this fragmentary dentition shows a well-developed, elongated pulp cavity and the relative thickness of the enameloid (acrodin) and circumpulpar dentine layers (Supplementary Fig. S5a, b). Under special lighting conditions (i.e., strong, laterally oriented light beam), this polished section shows thin and straight tubules penetrating from the dentine into the acrodin (Supplementary Fig. S6). Several incremental growth lines can also be observed in the dentine layer (Supplementary Fig. S6). The posterior half of this tooth (Supplementary Fig. S5c) was etched a few minutes with HCl and subsequently observed under scanning electron microscope (SEM). The acrodin layer has an inner portion consisting of fibres

more or less perpendicular to the dentine layer. The outer portion of the acrodin layer consists of woven fibre bundles which become densely arranged approaching to the crown surface (Supplementary Fig. S5d–f). The crown surface shows strongly woven fibres (Supplementary Fig. S5g). Similar acrodin organization and fibre bundle pattern have already been described in pycnodontiforms such as *Proscinetes* (see ref. [8]: fig. 54).

MHNM KHG 161 is a fragmentary vomer (38 mm in preserved length) with eight teeth of the left row preserved. The anteriormost tooth corresponds to the reduced, subconical tooth morphotype.

MHNM KHG 160 (Supplementary Fig. S1e, f) is a fragmentary vomer (40 mm in preserved length) with seven teeth of the left row preserved. An anteroposterior ridge is present on the lateral surface of the bone.

OCP DEK-GE 702 is a fragmentary right prearticular (59 mm in preserved length). Most of the symphysial region and eight teeth are preserved.

MHNM KHG 159 (Fig. 4a and Supplementary Fig. S2a–c) is a nearly complete right prearticular (52 mm in preserved length). The symphysial region and 10 teeth are preserved (nine labiolingually compressed teeth and one anterior subconical tooth). Two additional teeth are broken at the crown/root boundary in the anteriormost region of the row. The surface of the symphysial area shows a rugose texture. A shallow depression on the anterior face of the bone corresponds to the contact area with the dentary. The coronoid process is not preserved.

MHNM KHG 158 (Fig. 1e and Supplementary Fig. S2f–h) is a nearly complete right prearticular (72 mm in maximum preserved length). The coronoid process and 11 teeth are preserved (all labiolingually compressed teeth). The coronoid process is relatively low, with a short neck and a dorsal border markedly rounded. In dorsal view, it is strongly mediolaterally compressed (Supplementary Fig. S2h). The ventral margin is partly broken. The symphysial region is not preserved.

MHNM KHG 157 (Supplementary Fig. S2k–n) is a nearly complete right prearticular (63 mm in preserved length) with 11 teeth preserved (all labiolingually compressed teeth). An additional tooth is broken at the crown/root boundary in the anteriormost region of the row. A small, subconical vestigial tooth is also present on the medial wall of the bone, 4 mm below the third anterior tooth (as preserved) of the row. This tooth shows a small vertical wear facet on its lingual face. The symphysial region and the coronoid process are not preserved.

MHNM KHG 156 (Supplementary Fig. S2o) is a nearly complete left prearticular (66 mm in preserved length) with 13 teeth preserved (12 labiolingually compressed teeth and one

anterior subconical tooth). This specimen is still included in matrix and seen in lingual view. The symphysial region and the coronoid process are not preserved.

MHNM KHG 155 (Supplementary Fig. S2d, e) is a nearly complete left prearticular (40 mm in preserved length) with 10 teeth preserved (nine labiolingually compressed teeth and one anterior subconical tooth). The ventral margin is preserved. The symphysial region and the coronoid process are not preserved. The lateral face of the prearticular shows a low coronoid ridge and a shallow longitudinal depression for the insertion of the angular ventrolaterally.

MHNM KHG 152 (Figs 1c, d, 2f and Supplementary Fig. S2i, j) is a nearly complete left prearticular (86 mm in preserved length) lacking its ventral region and most of the coronoid process. The tooth row as well as the symphysial region are well preserved. 13 teeth are preserved (12 labiolingually compressed teeth and one anterior subconical tooth), and at least two additional teeth are broken at the crown/root boundary in the anteriormost region of the row. The highest teeth are located in the middle part of the row and are 8 mm high. The surface of the symphysial area shows a rugose texture. A shallow depression on the anterior face of the bone corresponds to the contact area with the dentary.

MHNM KHG 113 is a fragmentary left prearticular (46 mm in preserved length). The symphysial region and five teeth are preserved.

Size estimate. Based on the correlation existing between the prearticular length and the standard length of *Gyrodus* [29], the standard length of *Serrasalmimus* can be estimated using the largest known prearticular of this species (MHNM KHG 152). This nearly complete specimen would indicate a standard length of 700–750 mm. Given that the tail length is about one third of the standard length in *Gyrodus* [29], it can be assumed that the total length of *Serrasalmimus* may have reached between 930 and 1000 mm.

Distribution

All the specimens of *Serrasalmimus secans* gen. et sp. nov. come from the Paleocene and Eocene of the Sidi Chenanne and Sidi Daoui area, eastern part of the Ouled Abdoun Basin, Province of Khouribga, Morocco: lower bone bed of the phosphorite Bed IIa, late Selandian (Paleocene) in age; upper bone bed of the phosphorite Bed IIa, early Thanetian (Paleocene) in age; bone bed of the Intercalary phosphorite Bed II/I, ?late Thanetian–earliest Ypresian (?Paleocene–Eocene) in age; possibly phosphorite Bed I, early Ypresian (Eocene) in age [14,30] (Supplementary Table S1).

Serrasalmimus, known by several specimens, seems to be an endemic genus from the Ouled Abdoun Basin. It provides further evidence that the Moroccan phosphate basins represent a hot spot of vertebrate palaeobiodiversity at the K/Pg turnover and that their faunas are characterized by high endemism [31]. Interestingly, these faunas includes taxa that display trophic specializations and adaptations unique among their respective groups, exemplified by *Ocepechelon* within sea turtles [31] and *Serrasalmimus* within bony fishes.

Part B. Description and distribution of *Eoserrasalmimus cattoi* gen. et sp. nov.

Description of the specimen studied

MHNM KHG 165 (holotype and only known specimen; Fig. 2c and Supplementary Fig. S3a–d) is a nearly complete vomer (40 mm in preserved length) with most of the dentition preserved. The right side of the bone shows nine teeth (seven labiolingually compressed teeth and two anterior subconical (mammiform), monocuspid teeth with a slight cingulum) in the main, lateralmost row and eight smaller, ovoid teeth in the secondary row. The left side of the bone shows eight teeth (seven labiolingually compressed teeth and one anterior subconical (mammiform), bicuspid tooth with a slight cingulum) in the main row and six smaller, ovoid teeth (plus one broken tooth) in the secondary row. These secondary rows start posteriorly between the second and third posteriormost teeth of the main rows and converge anteriorly at the level of the posteriormost subconical teeth of the main rows. The labiolingually compressed teeth of the main rows show a crown with a rounded linguobasal bulge and two blunt, low cusps that tend to disappear in anterior teeth, probably due to functional wear. In the anterior portion of the dentition, a small subconical tooth is located where the two secondary rows converge between the posteriormost subconical teeth of the main rows. The acrodin canal system opening to the crown surface can be observed in most of the teeth, possibly due to a slight post-mortem abrasion of this specimen. In ventral view, the triangular-shaped edentulous area covers most of the medial and posterior region. This area is strongly depressed posteriorly.

Distribution

Eoserrasalmimus cattoi is known by a single specimen recovered from the phosphorite Bed III, late Maastrichtian in age, eastern part of the Ouled Abdoun Basin, Province of Khouribga, Morocco [14,30].

Part C. Description and distribution of *Damergouia lamberti* gen. et sp. nov.

Description of the specimens studied

MNHN.F.HGS176 (holotype; Fig. 2d and Supplementary Fig. S3e–m) is a complete left prearticular (27 mm in length) with nearly complete dentition. The bone is triangular in lateral view. The dentition shows two main rows of labiolingually compressed, triangular, elevated monocuspid teeth. A few additional, smaller teeth are also present. The lateral main row consists of five teeth with a well-developed lingual wear facet and an apex slightly recurved. The medial main row consists of four teeth with an apex slightly bent anteriorly. The anterior portion of the prearticular dentition consists of about twenty, irregularly arranged, small subconical teeth with a basal cingulum developing all around the crown. The medial face of the prearticular shows a well-developed, flattened, triangular edentulous shelf between the toothed area and the ventromedial margin. The symphysis is reduced in length, restricted to the anterior part of the prearticular (about half the prearticular length). The symphysis area is subtrapezoidal in shape, oblique in medial view. The lateral face of the prearticular shows a salient, curved coronoid ridge and a well-developed triangular depression for the insertion of the angular. The coronoid process is relatively low, robust and broad mediolaterally.

MNHN.F.HGS177 is a fragmentary vomer (about 12 mm in preserved width) with four teeth preserved. As indicated by preserved teeth and bases of broken ones, the dentition originally consisted of five rows of ovoid to subconical teeth ornamented with a finely granulated basal cingulum and coarse apical tubercles and wrinkles. The teeth of the medial and lateralmost rows are more developed that those of the two intermediate rows. A large wear facet, slightly oblique, is present on the labial face of one of the two preserved teeth (i.e., the posterior one) of lateralmost row.

Size estimate. Based on the correlation existing between the prearticular length and the standard length of *Gyrodus* [29], the standard length of *Damergouia* can be estimated using the only known prearticular of this species. This specimen would indicate a standard length of about 240 mm. Given that the tail length is about one third of the standard length in *Gyrodus* [29], it can be assumed that the total length of *Damergouia* may have reached 320 mm.

Distribution

Damergouia lamberti is known by only two specimens (originally referred to *Gyrodus* aff. *cretaceus*; see ref. [15]) recovered from the Turonian deposits of Tanout, Damergou area, Niger [15,16]. In a review of the fossil fishes of Africa, a Campanian age was erroneously assigned to all the vertebrate material from the Damergou area [32].

Part D. Revised diagnosis and distribution of *Polygyrodus cretaceus* (Agassiz, 1843)

Polygyrodus White, 1927

Type and only known species: *Gyrodus cretaceus* Agassiz, 1843

Polygyrodus cretaceus (Agassiz, 1843)
(Fig. 2a, b and Supplementary Fig. S3n–q)

Synonymy and most relevant bibliography:
? 1833 *Sphaerodus mammillaris* Agassiz: p. 15 (name only, *nomen nudum*) [33].
1839 *Gyrodus cretaceus* Agassiz: pl. 69a, fig. 13 (figure only, *nomen nudum*) [34].
1843 *Gyrodus cretaceus* Agassiz: p. 233, pl. 69a, fig. 13 [35].
? 1843 *Gyrodus mammillaris* Agassiz: p. 236, pl. 73, figs 1, 2 (name and figure only, *nomen nudum*) [35].
1850 *Gyrodus cretaceus* Agassiz; Dixon, p. 370, pl. 30, fig. 15 [36].
1850 *Gyrodus conicus* Dixon: p. 370, pl. 32, fig. 8 [36].
? 1850 *Gyrodus*, new species?; Dixon, p. xiii, pl. 32*, fig. 6 [36].
? 1870 *Gyrodus dixoni* Ooster *in* Ooster & Fischer-Ooster: p. 46, pl. 9, fig. 7 (*nomen dubium*) [37].
1909 *Gyrodus* (?) *cretaceus* Agassiz (*partim*); Woodward, p. 167, pl. 35, fig. 5, 6, *non* fig. 7 [38].
1912 *Gyrodus* (?) *cretaceus* Agassiz; Woodward, p. 251, pl. 54, fig. 5 [39].
? 1927 "*Gyrodus*" *bennetti* White: p. 189, fig. 2 [10].
1927 *Polygyrodus cretaceus* (Agassiz); White, p. 191 [10].
2002 *Polygyrodus cretaceus* (Agassiz); Longbottom & Patterson, p. 323, pl. 63, fig. 2 [11].

Revised diagnosis

Large-sized serrasalmimid pycnodontiform fish characterized by a crushing-type dentition and distinguished by the following unique combination of characters (autapomorphies marked with an asterisk): 1) teeth elevated, more or less conical, rugose, and bearing a basal cingulum (mammiform teeth)*; 2) teeth with a relatively narrow size range; 3) teeth of main rows slightly longer than wide; 4) vomerine dentition with three main rows separated by smaller, irregularly arranged teeth; 5) lateral rows more extended posteriorly that the medial row*; 6) medial row with teeth decreasing in size posteriorly*; 7) prearticular dentition elongated and relatively narrow; 8) prearticular dentition showing four or five badly defined rows of irregularly arranged teeth*; 9) short symphysis, about half the prearticular length; 10) coronoid process low and relatively broad anteroposteriorly.

Remark. As originally noted by Woodward [39], the dentary teeth seem to be preserved in NHMUK PV P 11157, a nearly complete lower dentition from the English Chalk still embedded in matrix (Fig. 2b and Supplementary Fig. S3p). The supposed dentary teeth (four on the right side and three on the left side) are slightly larger than the adjoining teeth present in the anteriormost portion of the specimen. However, their morphology (i.e., occlusal contour rounded to oval, crown elevated, wide basal cingulum) is very similar to that of the prearticular teeth, making their identification as dentary teeth uncertain. Therefore, this tentative observation is not taken into account in our phylogenetic analysis (see part E).

Size estimate. Based on the correlation existing between the prearticular length and the standard length of *Gyrodus* [29], the standard length of *Polygyrodus* can be estimated using the largest and most complete prearticulars of this species (NHMUK PV P 11157). This specimen would indicate a standard length of about 720 mm. Given that the tail length is about one third of the standard length in *Gyrodus* [29], it can be assumed that the total length of *Polygyrodus* may have reached 960 mm. This estimated total length of about 1 m equals that found for *Serrasalmimus*.

Distribution

Polygyrodus cretaceus is known from the Turonian (Late Cretaceous) of England [11,36,38–40], France (R.V. unpublished data), Germany [41], Czech Republic [42,43], and possibly Switzerland [37]. Some historical (19th Century) specimens from the English Chalk are labelled as from the ‘Lower Chalk’ or ‘Upper Chalk’, suggesting that this species may occur from the

Cenomanian to the Campanian. Isolated teeth of *Polygyrodus* sp. have been reported from the Maastrichtian of the Volga Region, Russia [44].

Part E. Phylogenetic analysis

Because osteological and histological characters demonstrate that *Serrasalmimus secans*, *Eoserrasalmimus cattoi*, *Damegouia lamberti* and *Polygyrodus cretaceus* are all pycnodontiforms, our phylogenetic analysis is designed to establish their interrelationships within the clade. We used Poyato-Ariza and Wenz's (2002) data matrix [18] with updates for new taxa and subsequent studies [45–48]. Some definitions and state orderings have been altered in order to fit with the new set of taxa. Characters 105–111 were added in order to address the conditions in the new taxa. The data were analysed using PAUP* 4.0b10 [49]. The tree resulting from this analysis is shown in Supplementary Fig. S4, with uniquely derived character states given along the branches corresponding to the new taxa.

Tree description:

Unrooted tree(s) rooted using outgroup method

Optimality criterion = parsimony

Character-status summary:

Of 111 total characters:

2 characters are of type 'ord' (Wagner)

109 characters are of type 'unord'

All characters have equal weight

2 characters are constant

18 characters are parsimony-uninformative

Number of parsimony-informative characters = 91

Gaps are treated as "missing"

Multistate taxa interpreted as uncertainty

Character-state optimization: Accelerated transformation (ACCTRAN)

Tree 1 (rooted using user-specified outgroup)

Tree length = 569

Consistency index (CI) = 0.5114

Homoplasy index (HI) = 0.4886

CI excluding uninformative characters = 0.4871

HI excluding uninformative characters = 0.5129

Retention index (RI) = 0.4991

Rescaled consistency index (RC) = 0.2553

→ One tree of 569 steps

List and definitions of characters (partly based on Poyato-Ariza and Wenz [18] and Ebert [48]):

1. **Body shape:**
   Char. 1 of P-A & W, states 0 and 2 inverted.
   0. discoid, 70-100%
   1. intermediate
   2. fusiform, less than 40%
   3. deep, more than 100%

2. **Relative position of dorsal apex:**
   Char. 2 of P-A & W, states 0 and 1 inverted.
   0. before point of insertion of dorsal fin
   1. dorsal prominence absent
   2. in point of insertion of dorsal fin

3. **Morphology of dorsal prominence:**
   Char. 3 of P-A & W, not modified (states 2 and 3 present in basal forms).
   0. dorsal prominence absent
   1. curved, dorsally oriented
   2. obtuse angle
   3. curved, anteriorly oriented
   4. curved, dorsally oriented

4. **Relative position of ventral apex:**
   0. apex absent
   1. before point of insertion of anal fin
   2. in point of insertion of anal fin

5. **Mouth gape:**
   0. horizontal or subhorizontal
   1. inclined
   2. subvertical, opening downward

6. **Prognathism:**
   0. absent
   1. present by elongation of mesethmoid, vomer and prearticular
   2. present by expansion of premaxilla and dentary

7. **Caudal pedicle:**
   Char. 7 of P-A & W, states 0 and 1 inverted.
   0. not differentiated
   1. differentiated

8. **Morphology of frontal bones:**
   0. curved and short
   1. concave and expanded

9. **Prefrontal bones:**
0. absent
1. present

10. **Frontal spines:**
0. absent
1. present, simple
2. present, compound

11. **Dermocranial fenestra:**
0. absent
1. present

12. **Parietal:**
Character 12 of P-A & W, not modified (state 1 present in basal forms).
0. single
1. divided
2. absent

13. **Parietal process:**
0. absent
1. present

14. **Supraoccipital spine:**
0. absent
1. present, simple
2. present, compound

15. **Extrascapulars hypertrophied:**
0. no
1. yes

16. **Extrascapular(s) fused to parietal:**
0. no
1. yes

17. **Endocranium largely exposed posteriorly:**
0. no
1. yes

18. **Anterior portion of infraorbital sensory canal:**
0. closely surrounding orbit
1. descending towards ethmoid region

19. **Infraorbitals:**
Char 21 of P-A & W, not modified (thus recoded in Ebert's matrix).
0. row of plates around the ventral and posterior border of the orbit
1. mosaic of small plates partially covering the cheek
2. educed to tubular ossifications around the infraorbital sensory canal
3. anterior infraorbital enlarged

20. **Infraorbital ornamentation:**
0. present in all infraorbitals
1. present only in posteriormost one
2. absent in all infraorbitals

21. **Suborbitals:**
Char. 23 of P-A & W, not modified. State 0 is added, state 1 becomes 2 in Ebert.
0. one or several rows

1. mosaic of small plates
2. absent as independent ossifications

22. Preopercular and hyomandibular:

Char. 24 of P-A & W, not modified. Two new states are added in Ebert.

0. preopercular single, smaller than opercular, hyomandibular deep, unornamented
1. preopercular single, hypertrophied, hyomandibular deep, unornamented
2. one large preopercular plus a small ornamented plate over the head of the hyomandibular
3. one large preopercular in close contact with a small ornamented portion of the hyomandibular, at the same superficial level
4. preopercular of similar size to expanded superficialornamented portion of hyomandibular

23. Condyle in articular head of hyomandibular:

0. absent
1. present

24. Opercular bone:

Char. 28 in P-A & W, state 0 added from Ebert.

0. well developed
1. reduced
2. extremely reduced

25. Branchiostegal rays:

Char. 30 of P-A & W, not modified, state 0 in outgroup. Ebert defined a state 2 for three rays, but it corresponds to state 0 of P-A & W.

0. more than two
1. two, relatively large, in contact
2. two, thin, separated

26. Morphology of premaxillary and dentary teeth:

Char. 32 in P-A & W, state 0 added from Ebert. State 4 added for Akromystax.

0. small, triangular to conic
1. robust, columnar to hookshaped
2. robust, barely incisiform
3. very flattened, fully incisiform
4. molariform

27. Number of premaxillary teeth:

Char. 32 in P-A & W, state 0 added from Ebert. States inverted. State 3 added, as well as state 4 for the presence of one tooth (states coded in Ebert's matrix, but not defined in his list of characters).

0. more than three
1. three
2. two
3. at least 8, arranged in at least two rows
4. one

28. Maxilla:

Char 35 of P-A & W, not modified. State 6 added for Polazzodus *(coded 5 in Ebert, 2015, but not defined).*

0. tooth-bearing, ornamented, elongated
1. edentulous, ornamented, ovoid
2. edentulous, ornamented, elongated
3. edentulous, unornamented, reniform
4. edentulous, unornamented, straight oral border
5. edentulous, unornamented, elongated oval
6. axe blade morphology

29. Morphology of vomerine teeth:

Char 36 of P-A & W, not modified. States 5 added for Akromystax. *State 6 added.*

0. villiform to conic

1. circular to subcircular contour
2. oval contour
3. reniform contour
4. triangular contour
5. oval, very elongated
6. labiolingually compressed (mono- or bicuspid)

30. Arrangement of vomerine teeth in regular rows:
Char 37 of P-A & W, not modified.
0. absent
1. present
2. absent anteriorly, present posteriorly

31. Number of vomerine tooth rows:
Char 38 of P-A & W, not modified. States 4 and 5 added. All states rearranged.
0. not arranged in rows
1. 6
2. 5
3. 4
4. 3
5. 2

32. Number of teeth in principal vomerine tooth row:
Char 39 of P-A & W, not modified. States ordered.
0. teeth not arranged in rows
1. seven or less
2. eight or nine
3. 10 or more

33. Alternation of teeth on main vomerine tooth row:
0. absent
1. present

34. Number of dentary teeth:
Char 42 of P-A & W, not modified.
0. more than five
1. 5
2. 4
3. 3
4. 2

35. Morphology of prearticular teeth:
State 0 added (outgroup). Char. recoded for basal forms. State 6 added.
0. villiform to conic
1. oval contour
2. circular (to sub-circular) contour
3. sigmoid to drop-shaped contour
4. extremely elongated in contour, long axis perpendicular to row axis
5. oval, elongated, long axis of teeth coincident with row axis
6. labiolingually compressed (mono- or bicuspid)

36. Arrangement of prearticular teeth in regular row(s):
Char 44 of P-A & W, not modified.
0. absent
1. present
2. absent anteriorly, present posteriorly

37. Number of prearticular tooth row(s):
Char 45 of P-A & W, not modified. State 5 added.
0. not arranged in rows

1. 5-6
2. 4
3. 3
4. 2
5. 1

38. Number of teeth on main prearticular tooth row:
Char 46 of P-A & W, not modified. States ordered.
0. teeth not arranged in rows
1. seven or less
2. eight or nine
3. 10 or more

39. Coronoid process:
Char 46 of P-A & W, not modified (states 0 and 1 inverted in Ebert, 2015).
0. low, curved
1. high, straight dorsal border
2. high, club shaped
3. low, straight dorsal border

40. Crenulations/cuspidation in vomerine and prearticular teeth:
Char 49 of P-A & W, not modified. State 3 added
0. absent
1. crenulation occasionally present, weak
2. crenulation present in most teeth, strong
3. one or two cuspids

41. Groove on vomerine and prearticular teeth:
0. absent
1. present

42. Number of vertebrae:
Char 52 of P-A & W, not modified
0. 35 or more
1. 30-34
2. 25-29
3. 24 or less

43. Neural and haemal corresponding arcocentra:
0. not surrounding notochord
1. surrounding notochord partially
2. surrounding notochord completely

44. Neural and haemal adjacent arcocentra:
Char 54 of P-A & W, not modified. State 0 added to Ebert (2015).
0. separated from each other
1. simple contact
2. complex contact
3. hyper-complex contact
4. expanded and imbricate

45. Sagittal flanges on neural and haemal spines:
Char 55 of P-A & W, not modified. State 0 and 4 added to Ebert (2015).
0. absent
1. anterior, small and short
2. anterior, large and long
3. anterior and posterior
4. anterior and posterior with strengthened margins

46. Number of autogenous anterior neural spines:

Char 56 of P-A & W, but state for outgroup (?) deleted. State 1 becomes 0.

0. most of them, including caudal ones
1. 10 or more
2. seven to 10
3. six or less

47. Relative length of last neural spine not supporting precurrent caudal fin rays:

0. reduced
1. less than half as long as preceding spines
2. vestigial

48. Number of epichordal elements of caudal endoskeleton:

0. nine or more
1. six to eight
2. four or five
3. three or fewer

49. Relative development of hypochordal elements of caudal endoskeleton:

0. only slightly enlarged
1. enlarged, plate-like
2. one hypertrophied element
3. two hypertrophied elements

50. Number of hypochordal elements of caudal endoskeleton:

Char 60 of P-A & W, not modified. State 0 added to Ebert (2015), states 0 and 3 inverted.

0. 14 or more
1. 12-13
2. 9-11
3. six to eight

51. Diastema:

0. absent
1. present

52. Cleithrum:

Char 62 of P-A & W, not modified. States 0 and 1 added to Ebert (2015).

0. two limbs in angle, anteroventral limb subhorizontal
1. curved, anteroventral limb subhorizontal, slightly expanded
2. curved, anteroventral limb subvertical, expanded
3. cleithrum with three limbs
4. cleithrum with four limbs

53. Spines on cleithrum:

0. none
1. 1, hypertrophied
2. about 10
3. about 50

54. Position of pelvic fins (ratio prepelvic distance/standard length):

0. 45-55%
1. more than 55%
2. less than 45%

55. Position of dorsal fin (predorsal length/standard length):

Basal forms recoded.

0. 82% and more
1. 68-81%
2. 59-67%
3. 50-58%
4. less than 49%

56. **Number of dorsal axonosts:**

0. 30-39
1. fewer than 20
2. 20-29
3. 40-47
4. 48-59
5. 60 or more

57. **Dorsal axonost not supporting lepidotrichium (free axonost):**

0. absent
1. present

58. **Morphology of dorsal fin:**

Char 68 of P-A & W, not modified.

0. strip-like
1. falcate to acuminate
2. sigmoid outline
3. rounded in center
4. rounded anteriorly
5. square

59. **Position of anal fin (preanal length/standard length):**

0. 50%-59%
1. 60%-69%
2. 70%-79%
3. 80%-89%

60. **Number of anal axonosts:**

0. 20-27
1. 10-19
2. 28-34
3. 35-46
4. 47 or more
5. 9 or fewer

61. **Urodermals:**

Char 71 of P-A & W, not modified.

0. not differentiated
1. a series of three or more
2. two
3. one
4. absent

62. **Number of caudal principal fin rays:**

Char 72 of P-A & W, not modified.

0. 20-25
1. nine or less
2. 10-19
3. 26-35
4. 36 or more

63. **Morphology of caudal fin:**

Char 73 of P-A & W, not modified.

0. outgroup
1. stalked
2. distal border convex
3. distal border concave
4. distal border straight
5. double emarginated

6. vertical

64. Ossification of scales:

0. complete in all scales
1. complete in abdominal scales, incomplete in caudal scales
2. complete in ventral scales and in some dorsal scales
3. complete in ventral scales, incomplete in dorsal scales
4. incomplete in all scales
5. scales absent

65. Distribution of scales:

Char 76 of P-A & W, not modified. States 0 and 3 inverted in Ebert (2015).

0. whole body
1. whole body except caudal pedicle
2. abdominal region plus part of the caudal region
3. only abdominal region
4. body naked

66. Arrangement of scales:

0. rows in same direction
1. rows in different directions
2. not forming rows
3. scales absent

67. Ornamentation of scales:

0. tubercles
1. ridges
2. reticulation
3. small spines
4. smooth

68. Large spines on scales:

0. none
1. one
2. several

69. First dorsal ridge scale:

Char 86 of P-A & W, not modified. State 0 added to Ebert (2015).

0. not differentiated
1. about same size than subsequent ridge scales
2. larger than subsequent ridge scales
3. absent

70. Scutellum-like contour scales:

0. absent
1. present, dorsal only
2. present, ventral only
3. present, dorsal and ventral
4. contour scales absent

71. Number of differentiated dorsal ridge scales:

0. dorsal contour scales not differentiated
1. 18 or more
2. 15 to 17
3. 10 to 14
4. seven to nine
5. one or two
6. dorsal contour scales absent

72. Arrangement of dorsal ridge scales:

0. dorsal contour scales in close contact with each other
1. point contact
2. separated from each other
3. dorsal contour scales absent

73. Number of spines on dorsal ridge scales:
Char 90 of P-A & W, not modified.
0. no spines on dorsal contour scales
1. one or two
2. three or four
3. five or more
4. midline serrated
5. dorsal contour scales absent

74. Distribution of spines on dorsal ridge scales:
Char 91 of P-A & W, not modified. States 0 and 1 inverted in Ebert (2015).
0. no spines on dorsal contour scales
1. all along the midline, or centered if only one spine present
2. posterior region (at most two thirds) of the midline
3. anterior region (at most two thirds) of the midline
4. dorsal contour scales absent

75. Contact of spines on each dorsal ridge scale:
Char 92 of P-A & W, not modified. States 0 and 1 inverted in Ebert (2015).
0. no spines on dorsal contour scales
1. separated from each other
2. in contact with each other
3. dorsal contour scales absent

76. Relative size of anterior and posterior spines on each dorsal ridge scale:
Char 93 of P-A & W, not modified. States 0 and 1 inverted in Ebert (2015).
0. no spines on dorsal contour scales
1. similar size
2. spines of increasing size in cephalocaudal sense
3. dorsal contour scales absent

77. Number of ventral keel scales:
Char 94 of P-A & W, not modified. States 0 added to Ebert (2015).
0. not differentiated
1. 22 or more
2. 18 to 21
3. 15 to 17
4. 10 to 14
5. two or three
6. ventral keel scales absent

78. Arrangement of ventral keel scales:
0. close contact with each other
1. point contact
2. ventral keel scales absent

79. Maximal number of spines on ventral keel scales of adult specimens:
Char 96 of P-A & W, not modified.
0. no spines on ventral keel scales
1. one to three
2. four to six
3. seven or more
4. ventral keel scales absent

80. Distribution of spines on ventral keel scales:

Char 97 of P-A & W, not modified. States 0 and 1 inverted in Ebert (2015).
0. no spines on ventral keel scales
1. all along the midline, or centered if only one spine present
2. posterior region (at most two thirds) of the midline
3. ventral keel scales absent

81. Contact of spines on each ventral keel scale of adult specimens:
Char 98 of P-A & W, not modified. States 0 and 1 inverted in Ebert (2015), state 4 added.
0. no spines on ventral keel scales
1. separated from each other
2. in contact with each other
3. ventral keel scales absent
4. imbricate

82. Relative size of anterior and posterior spines on each ventral keel scale of adult specimens:
0. no spines on ventral keel scales
1. all spines of similar size
2. spines of increasing size in cephalocaudal sense
3. ventral keel scales absent

83. Several scales attached to the contour scales:
0. no
1. yes
2. contour scales absent

84. Number of post-cloacal ventral keel scales:
0. cloacal and contour scales not differentiated
1. 10 or more
2. seven or eight
3. five or six
4. three or four
5. two
6. one
7. none

85. Number of anterior cloacal modified scales:
Char 102 of P-A & W, not modified. State 5 added.
0. cloacal scales not modified
1. mosaic of little scales
2. two
3. one
4. cloacal scales absent
5. 3

86. Number of posterior cloacal modified scales:
Char 103 of P-A & W, not modified. State 6 added.
0. cloacal scales not modified
1. mosaic of little scales
2. three
3. two
4. one
5. no scales, posterior part of anal notch supported by a rib
6. cloacal scales absent

87. Bifid scale in cloaca:
0. absent
1. present
2. present plus several comma-shaped scales

88. Post-cloacal notch:

0. absent
1. present

89. Supracloacal scale:
0. absent
1. present, contacting only cloacal scales
2. present, contacting also non-differentiated scales adjacent to cloacal scales

90. First ventral keel scale:
0. smaller than subsequent scales
1. larger than subsequent scales
2. ventral keel scales absent

91. Fringing fulcra between the basal fulcra as well as between the basal fulcra and the first segmented ray of the dorsal and anal fin:
0. present
1. absent

92. Ethmoid commissure (a sensory canal between the infraorbital canal and the premaxilla):
0. absent
1. present

93. Complete scale rows posterior to the cloaca:
0. one
1. two
2. three to four
3. five to six
4. more than six
5. zero

94. Dermal supraoccipital:
Char. 15 of P-A & W, deleted in subsequent analyses because present only in basal forms.
0. absent
1. single
2. divided into two or more unpaired plates

95. Opercular process of hyomandibular:
Char. 25 in P-A & W, only present in basal genera.
0. present, well developed
1. present, reduced
2. absent

96. Ossifications in gular region:
Char. 29 in P-A & W, only present in basal genera.
0. large gular plate
1. small, numerous tesserae
2. no ossifications

97. Premaxillary process:
Char. 31 in P-A & W, only present in basal genera.
0. profound
1. anteriorly placed, long, superficial

98. Dentary:
Char. 41 in P-A & W, only present in basal genera.
0. well developed, relatively broad
1. small, posteriorly elongated and simple
2. small, posteriorly bifid

99. Central papilla in vomerine and prearticular teeth:

Char. 48 in P-A & W
0. absent
1. present

100. Fringing fulcra:
Char. 74 in P-A & W
0. present, numerous
1. present, scarce
2. absent

101. Scale rows:
Char. 79 in P-A & W, only present in basal genera.
0. simple
1. double

102. Scale rows between the bases of the lepidotrichia of the dorsal and anal fins:
Char. 80 in P-A & W, only present in basal genera.
0. absent
1. present

103. Nuchal plates:
Char. 83 in P-A & W, only present in basal genera.
0. absent
1. present

104. Contour scales:
0. not differentiated
1. differentiated
2. absent

105. Prearticular symphysis:
New character.
0. > 2/3 length of the prearticular
1. between 1/3 and 2/3 the length of the prearticular
2. < 1/3 length of the prearticular

106. Prearticular and vomerine teeth elevated:
New character.
0. absent
1. present

107. Linguobasal cingulum in main prearticular and vomerine teeth:
New character.
0. absent
1. present

108. Dentition type:
New character.
0. crushing
1. crushing/cutting
2. cutting

109. Mammiform teeth with cingulum all around the crown:
New character.
0. absent
1. present in most of the dentition
2. present anteriorly, absent posteriorly

110. Ventral surface of the vomer (buccal roof):
New character.

0. plane or convex
1. concave in cross section

111. Ventral surface of the vomer :
New character.
0. completely toothed
1. partially toothed
2. completely edentulous

Data matrix:

See Supplementary Table S2.

Part F. Carnivorous specialization of the Serrasalmimidae

The serrasalmimid dentitions show a spectacular carnivorous specialization, which is most advanced in the genus *Serrasalmimus.* The most remarkable carnivorous features are:

- Enlargement and predominance of the lateralmost tooth row with specialized carnivorous function, at the expense of the internal tooth rows that show a trend for reduction in the family;
- Development of large and sharp teeth which are laterally compressed, slightly recurved. These teeth show an overlapping arrangement. The high and blade-like crown shows one or two main cusps;
- Sharp teeth with enhanced shearing function as indicated by the development of strong attritional wear facets onto the shearing side of the occluding crests of opposing teeth (i.e., a wear facet on the labial side of upper teeth and on the lingual side of lower teeth). There may be one or two wear facets (of slightly different inclination) in *Serrasalmimus* teeth. Several specimens show distinct (vertical and oblique) wear striae generated by opposed shearing crests that illustrate the jaw movements during tooth-to-tooth contact (Supplementary Fig. S2n);
- Presence of a linguobasal cingulum in prearticular teeth, which has the function to protect the gum from food or tooth contact during bite and occlusion, as seen in mammals (e.g., refs [50,51]). The functional role of this cingulum is indicated by the extension, in mature individuals, of the shearing wear facet onto it (cingulum truncated by the wear) (Supplementary Fig. S2n);
- Short symphysis of the prearticular (Fig. 4a).

Part G. Analogous trophic specialization in other fish groups

Serrasalmidae (Osteichthyes: Characiformes)

Three main clades are recognized within the Serrasalmidae: the pacu clade, the *Myleus* clade, and the piranha clade [22]. The fossil record of some modern genera (e.g., *Colossoma* and *Mylossoma*) dates back to the early Miocene [28,52], but the presence of various dental morphotypes in specimens from the latest Cretaceous and Paleogene similar to those observed in living forms suggests earlier divergence times and trophic diversification in the serrasalmid evolution [22]. The pacu clade (including *Colossoma* and *Mylossoma*) and the *Myleus* clade (including *Myleus* and *Tometes*) show crushing and crushing–cutting dentitions, respectively, with robust teeth arranged in two rows on the premaxilla [12,13,22,23,53–55] (Fig. 4 and Supplementary Fig. S7a, b), whereas the piranha clade (including *Pygocentrus* and *Serrasalmus*) shows in each jaw a single row of labiolingually compressed bicuspid and tricuspid teeth [13,22–24,53] (Fig. 4 and Supplementary Fig. S7c). Piranhas are clearly characterized by a cutting dentition. Their teeth are overlocked by the seating of the distal cusplets into mesial recesses present in the succeeding teeth, thus reinforcing the cohesion of the tooth row and improving the effectiveness of the cutting edge throughout the dentition [24]. A fossil genus, *Megapiranha*, has been recently described from the Miocene of Argentina and has been recovered as the sister taxon to the piranha clade [13]. The premaxilla of *Megapiranha* shows triangular teeth that are slightly compressed labiolingually and arranged in a shallow zig-zag row, a condition intermediate between the single tooth row of piranhas and the double tooth row of the pacu and *Myleus* clades [12]. Members of the pacu clade such as *Colossoma* use their powerful jaws bearing molariform teeth for crushing seeds and fruits [12]. Members of the *Myleus* clade such as *Myleus* and *Tometes* are more generalized herbivores, with dentitions suitable for crushing seeds and fruits and for cutting leaves and flowers; these fishes also feed on small invertebrates [12]. Members of the piranha clade such as *Pygocentrus* and *Serrasalmus* are predominantly carnivorous forms which prey mainly upon fishes, either swallowing whole individuals of small-sized species or biting pieces of flesh and fins of larger species [12,56,57].

Dalatiidae (Chondrichthyes: Squaliformes)

Among cartilaginous fishes, a similar foraging strategy is known among the Dalatiidae, especially in kitefin and cookiecutter sharks, *Dalatias* and *Isistius*, respectively. These dwarf to medium-sized specialized sharks have powerful jaws with a lower dentition consisting of a

single functional row of large, labiolingually compressed, triangular teeth [58], roughly similar to that of piranhas and *Serrasalmimus* (Supplementary Fig. S8). Teeth are imbricated and show an overlapping arrangement [59], like in serrasalmimids. However, the dignathic heterodonty is strongly developed and the upper jaw of these sharks has a clutching function. Cookiecutter sharks prey mostly upon small deep-water cephalopods and fishes, but are also known to regularly cut out flesh plugs from the body or fins of large pelagic fishes and marine mammals [60,61].

Part H. Comments on the taxonomic assignment of *Serrasalmimus*

It worth noting that the dentition of *Serrasalmimus* misleadingly resembles to some extent to those found in some reptiles. Thus, *Serrasalmimus* could be erroneously misinterpreted as a squamate. However, the various features which could be regarded as squamate-like characters correspond to superficial convergence. It is true that some squamates, such as teiid scincomorph lizards, roughly show a similar tooth crown morphology (i.e., bicuspid teeth). However, these teeth are pleurodont, spaced, and relatively small (unlike in *Serrasalmimus*) [62]. In squamates with a cutting dentition (e.g., agamid lizards), the upper dentition is labial to the lower dentition when mouth closed (unlike in *Serrasalmimus*) [63,64], and the organization of the enamel layer (i.e., parallel rods running out towards the tooth surface in the agamid spiny-tailed lizard *Uromastyx* [63]) is distinct from that of the acrodin layer (i.e., woven fibre bundles densely arranged) observed in *Serrasalmimus* teeth. The tooth-bearing bone of the lower jaw of squamates, corresponding to the dentary, shows a typical subdental self on its lingual surface and some foramina of its labial surface (unlike in *Serrasalmimus*) [65,66]. The posterior margin of the dentary of squamates shows a typical surangular sinus located between two (i.e., surangular and angular) processes [66] (all absent in *Serrasalmimus*). The shape of the coronoid process of the dentary of squamates (including amphisbaenians) is relatively small and pointed [65–67] (large and more rounded in *Serrasalmimus*). The vomer of *Serrasalmimus* might be erroneously misinterpreted as two fused maxillae. However, this condition is rarely observed in reptiles (known in a few pterosaurs and dinosaurs), and it seems (to our knowledge) that fused maxillae have never been reported in squamates. Furthermore, the dorsal surface of the vomer of *Serrasalmimus* shows the base of a broken median dorsal crest (typical of the pycnodontiform vomer [8,68]) that would invalidate this hypothesis.

Like in squamates, the apices of the teeth of *Serrasalmimus* are devoid of translucent acrodin caps. In pycnodonts, a translucent apical cap of acrodin can be observed in incisive-like teeth of the dentary and premaxilla, as well as in hook-shaped branchial teeth (dental types unknown in *Serrasalmimus*) (R.V. pers. obs.). This translucent acrodin cap was lost in molariform teeth of pycnodonts. The peculiar, sharp teeth of *Serrasalmimus* being derived from molariform teeth, the lack of translucent apical caps of acrodin simply indicates that a reversion of this character did not occur during the serrasalmimid evolution. It is also worth noting that several taxa of bony fishes with a cutting dentition have teeth devoid of translucent apical caps of acrodin (e.g., Saurodontidae; ref. [69]: fig. 4b).

Most of the synapomorphic characters defining the Pycnodontiformes in previous studies [5,8,18] cannot be observed in the material of *Serrasalmimus*. However, it is worth noting that this is due to the lack of the corresponding skeletal elements in our material and not to a "true" absence of these characters. Since vomers and prearticulars are the only skeletal elements known in serrasalmimids, the only synapomorphic characters that can be observed are "teeth on vomer and prearticular arranged in more or less regular rows" and "long, stout mandibular symphysis". Whereas the multiple tooth-rowed dentitions of *Polygyrodus, Damergouia* and *Eoserrasalmimus* can be clearly referred to the Pycnodontiformes (as other dentition-based pycnodont taxa; e.g., *Arcodonichthys* [70]), we understand that the peculiar, single-rowed upper and lower dentitions of *Serrasalmimus* could cause problems and questions regarding the taxonomic assignment of this fish. However, despite the derived condition of *Serrasalmimus*, several features observed in this genus are also found in pycnodont fishes [8], such as (psa: pycnodontiform synapomorphy, according to refs [71,72]):

- Small mammiform, *Gyrodus*-like teeth with a cingulum (present only in the anteriormost portion of the dentition);
- Structural organization of the acrodin layer (woven fibre bundles);
- Thin and straight tubules penetrating from the dentine into the acrodin;
- Single-layered acrodin comprising only one type of tubules ("acrodin canals of type A" *sensu* Ørvig 1978 [73]);
- Ankylothecodont-like tooth attachment;
- Upper (vomerine) tooth row occluding lingually to lower (prearticular) tooth row;
- Vomer single and median (psa);
- Median dorsal crest of the vomer (psa);
- Prearticular symphysis deep;
- Prearticular symphysial articulation rugose;

- Coronoid process arising from the posterolateral side of the prearticular (psa)
- Coronoid process stout, rounded in lateral view;
- Coronoid ridge on the lateral surface of the prearticular.

In addition, the presence of a vestigial tooth in the prearticular MNHM KHG 157 is the remnant of one of the medial tooth rows lost during serrasalmimid evolution. This unique combination of characters makes *Serrasalmimus* an unusual but unambiguous pycnodont fish which is placed as the closest relative of the Late Cretaceous genus *Eoserrasalmimus*.

It worth noting that mono-, bi- or multi-cuspidate teeth roughly similar to those of *Serrasalmimus* are also known in several Cenozoic teleostean fishes, such as *Gymnarchus* (Osteoglossiformes: Gymnarchidae) [74,75], characids (Characiformes) [76,77], cichlids and siganids (Perciformes) [78,79]. However, the teeth of *Gymnarchus* can be distinguished by several features (i.e., square-shaped crowns, finely serrated carinae, no basal cingulum). The teeth of the algae-scraping characids (Characiformes) [77], cichlids and siganids are very small (less than 1 mm in length), devoid of basal cingulum, and do not show clear wear facets [79]. Furthermore, the upper tooth row never occludes lingually to the lower tooth row in teleostean fishes (e.g., [23,78]), unlike in pycnodontiform fishes.

Like in durophagous pycnodontiforms, *Serrasalmimus* shows a typical woven pattern of acrodin bundles in the outer tooth layer (Supplementary Fig. S5g). This condition differs from crushing teeth of teleosts with an outer layer constituted of parallel fibre bundles [8]. Acrodin canals of type A (*sensu* Ørvig 1978 [73]) are present in *Serrasalmimus* (Supplementary Fig. S6). Ørvig [73] defined these canals as follows: “the acrodin canals of type A are thin and as a rule fairly straight. They penetrate for varying distances into the hard tissue from its basal boundary, sometimes reaching almost to its external surface, and they frequently lie in direct continuity with the dentinal tubules of the adjoining dentine”. This type is mainly present in pycnodontiforms (and in basal actinopterygians such as palaeonisciforms and polypteriforms) (see ref. [8], fig. 52 and ref. [73], fig. 63 for examples of pycnodont tooth sections showing a condition similar to that observed in the *Serrasalmimus* tooth section), but it seems to be absent in teleostean fishes [8,73]. In the latter, the acrodin layer is generally divided in two sublayers, and thus shows two co-occurring types of canals: 1) straight and parallel canals (acrodin canals of type C *sensu* Ørvig 1978 [73]), which are restricted to the external part of the layer, and 2) sinuous, irregularly arranged canals (acrodin canals of type B *sensu* Ørvig 1978 [73]), which are located in the internal part of the layer (see ref. [8], fig. 55, ref. [73], figs 27, 28 and ref. [80], figs 5, 10 for comparisons).

Serrasalmimus is described on the basis of isolated tooth-bearing elements that are identified as vomer and prearticular bones. The upper jaw bone cannot be interpreted as fused premaxillae because of the lack of anterior ascending processes. In lateral view, the vomer of *Serrasalmimus* shows a well-developed vertical oral border, with a slight anteroposterior ridge of the bone present above the tooth row (Supplementary Fig. S9a, b). This is very similar to the condition observed in some pycnodonts (e.g., *Gyrodus*, *Acrotemnus* [10,81], R.V. pers. obs.; Supplementary Fig. S9c–f). In most teleosteans, the vomer is unpaired and bears tooth whose location show a wide array of patterns. In most of these fishes, however, the vomer is very shallow, without lateral vertical border and without a dorsal medial crest (e.g., the osteoglossiform *Scleropages formosus* [82]; Supplementary Fig. S9g, h). In anguilliforms, the vomer is fused with the ethmoid complex to form the ethmovomer (e.g., *Moringua edwardsi* [83]; Supplementary Fig. S9i, j). This ossification is stout and bears very different kind of teeth depending of the species. Although this massive ossification might recall the vomer of serrasamimids, its organization is very different from the vomer of *Serrasalmimus* because it forms the tip of the snout including its dorsal side, and consequently it never bears a medial dorsal crest that inserts within other bones as in pycnodonts. Among anguilliform fishes, some pike congers such as *Muraenesox* and *Cynoponticus* (Muraenesocidae) have a vomer bearing large, triangular, laterally compressed teeth [84]. However, these principal vomerine teeth are arranged on a single row located medially and do not occlude with any teeth of the lower jaw. In addition to the main medial row, the lateral margins of the vomer bear a row of small teeth. This condition is clearly distinct from that found in *Serrasalmimus*.

In most non-teleost actinopterygians, the vomer is a paired ossification. However, there are a few exceptions, such as in some pachycormiforms. *Protosphyraena* is a Cretaceous pachycormiform with laterally compressed teeth and with an unpaired toothed rostrodermethmoid [85–87]. Here again, the tooth attachment and the mode of occlusion of the jaw, as well as the very different shape of the unpaired toothed element, which consists of a vomer bearing one pair of large paramedial teeth fused to a cylindrical rostrodermethmoid in *Protosphyraena* (Supplementary Fig. S9k, l), prevent any phylogenetic connections of the latter with *Eoserrasalmimus* and *Serrasalmimus*.

The lower jaw bone of *Serrasalmimus* cannot be interpreted as a dentary because of the features of the symphysis. The symphysial articulation is deep and rugose, like in the prearticular of most pycnodonts [7,8,72] (e.g., *Phacodus*; R.V. pers. obs.), but unlike in most teleostean fishes, in which it is often marked by a series of diagonal ridges [88,89]. The well-developed coronoid process, arising from the posterolateral side, is stout and shows a rounded

shape in lateral view. This condition is found in many pycnodonts [7,8,18,72] whereas the coronoid process of teleostean dentaries is not as developed [88]. The lower jaw elements of *Eoserrasalmimus* and *Serrasalmimus* show superficial resemblance to jaw bones of saurodontids (Cretaceous ichthyodectiforms), such as pointed teeth with cutting edges, a deep symphysis and a rounded coronoid process posteriorly located. However, the ankylothecodont-like tooth attachment, the absence of a well-defined fossa on the medial side of the mandible near its anterior extremity and the mode of occlusion indicated by the wear facet on teeth in *Eoserrasalmimus* and *Serrasalmimus* are incompatible with saurodontid features [90,91].

Posteriorly, the lower jaw bone of *Serrasalmimus* shows no articulation fossa, unlike teleostean dentaries. The latter show a deep sinus for the articulation with the articular [88,92]. Interestingly, upper and lower jaw bones of *Serrasalmimus* are both devoid of teeth in their anteriormost (frontal) portion, a feature that is characteristic of the vomerine and prearticular dentitions of pycnodonts. In the anteriormost portion of the jaws, upper and lower prehensile teeth were borne by the premaxillary and dentary bones, respectively, which are here unpreserved. Lastly, the lateral surface of lower jaw bone of *Serrasalmimus* shows no pores or grooves, unlike in teleostean dentaries. In the latter, these openings are related to the mandibular sensory canal (e.g., [89,91,92]). In pycnodonts, the mandibular sensory canal seems to run along the entire length of the jaw, but it has never been specifically described in the prearticular bone [8].

Part I. Supplementary references

29. Licht, M. The relationship of prearticular length and standard length in pycnodontiform fishes. *Stud. Geol. Salmant.* **45**, 139–148 (2009).

30. Yans, J. *et al.* First carbon isotope chemostratigraphy of the Ouled Abdoun phosphate Basin, Morocco; implication for dating and evolution of earliest African placental mammals. *Gondwana Res.* **25**, 257–269 (2014).

31. Bardet, N. *et al.* A giant chelonioid turtle from the Late Cretaceous of Morocco with a suction feeding apparatus unique among tetrapods. *PLoS ONE* **8**: e63586 (2013).

32. Murray, A. M. The Palaeozoic, Mesozoic and Early Cenozoic fishes of Africa. *Fish Fish.* **1**, 111–145 (2000).

33. Agassiz, L. *Recherches sur les Poissons Fossiles, 1st "Livraison", Vol. 2, Part I.* (Petitpierre, Neuchâtel, 1833).

34. Agassiz, L. *Recherches sur les Poissons Fossiles, 10th and 12th "Livraisons", Vol. 2, Part II* (Petitpierre, Neuchâtel, 1839).

35. Agassiz, L. *Recherches sur les Poissons Fossiles, 17th "Livraison", Vol. 2, Part II* (Jent and Gassmann, Soleure, 1843).

36. Dixon, F. *The Geology and Fossils of the Tertiary and Cretaceous Formations of Sussex* (Longman, Brown, Green, and Longmans, London, 1850).

37. Ooster, W.-A. & Fischer-Ooster, C. von. *Protozoe Helvetica, Vol. 2* (Haller, Bern, 1870–1871).

38. Woodward, A. S. The fossil fishes of the English Chalk, Part V. *Monogr. Palaeontogr. Soc. London* **63**, 153–184 (1909).

39. Woodward, A. S. The fossil fishes of the English Chalk, Part VII. *Monogr. Palaeontogr. Soc. London* **65**, 225–264 (1912).

40. Friedman, M., Beckett, H. T., Close, R. & Johanson, Z. in *Arthur Smith Woodward: His Life and Influence on Modern Vertebrate Palaeontology* (eds Johanson, Z., Barrett, P. M., Richter, M. & Smith, M.) 165–200, *Geol. Soc. London Spec. Publ.* **430** (2016).

41. Roemer, F. A. *Die Versteinerungen des Norddeutschen Kreidegebirges* (Hahn, Hannover, 1841).

42. Reuss, A. E. *Die Versteinerungen der Böhmischen Kreidefrormation*.(Schweizerbart, Stuttgart, 1845–1846).

43. Ekrt, B., Košťák, M., Mazuch, M., Voigt, S. & Wiese, F. New records of teleosts from the Late Turonian (Late Cretaceous) of the Bohemian Cretaceous Basin (Czech Republic). *Cret. Res.* **29**, 659–673 (2008).

44. Popov, Y. V. & Yarkov, A. A. A new giant species of *Edaphodon* (Holocephali: Edaphodontidae) from the Beryozovaya Beds (Lower Paleocene) of the Volgograd Volga Region. *Paleontol. J.* **35**, 183–187 (2001).

45. Poyato-Ariza, F. J. & Wenz, S. in *Mesozoic Fishes 3 – Systematics, Paleoenvironments and Biodiversity* (eds Arratia, G. & Tintori, A.) 371–378 (Verlag Dr. Friedrich Pfeil, 2004).

46. Poyato-Ariza, F. J. & Wenz, S. *Akromystax tilmachiton* gen. et sp. nov., a new pycnodont fish from the Lebanese Late Cretaceous of Haqel and en Nammoura. *J. Vertebr. Paleontol.* **25**, 27–45 (2005).

47. Machado, L. P. C. & Brito, P. M. The new genus *Potiguara* (Actinopterygii: Pycnodontiformes) from the Upper Cretaceous northeast Brazil. *J. Vertebr. Paleontol.* **26**, 1–6 (2006).

48. Ebert, M. The Pycnodontidae (Actinopterygii) in the Late Jurassic: 2) *Turboscinetes* gen. nov. in the Solnhofen Archipelago (Germany) and Cerin (France). *Archaeopteryx* **33**, 12–53 (2015).

49. Swofford, D. L. *PAUP*: Phylogenetic analysis using parsimony and other methods (software)* (Sinauer Associates, Sunderland, Massachusetts, 2000).

50. Mills, J. R. E. Development of the protocone during the Mesozoic. *J. Dent. Res.* **46**, 787–791 (1967).

51. Kermack, D. M., Kermack, K. A. & Mussett, F. The Welsh pantothere *Kuehneotherium praecursoris*. *J. Linn. Soc. (Zool.)* **47**, 407–423 (1968).

52. Dahdul, W. M. Fossil serrasalmine fishes (Teleostei: Characiformes) from the lower Miocene of north-western Venezuela. *Spec. Pap. Palaeontol.* **71**, 23–28 (2004).

53. Eigenmann, C. H. The Serrasalminae and Mylinae. *Ann. Carnegie. Mus.* **9**, 226–272 (1915).

54. Jégu, M. & Santos, G. M. Révision du statut de *Myleus setiger* Müller & Troschel, 1844 et de *Myleus knerii* (Steindachner, 1881) (Teleostei: Characidae: Serrasalminae) avec une description complémentaire des deux espèces. *Cybium* **26**, 33–57 (2002).

55. Jégu, M., Keith, P. & Belmont-Jégu, E. Une nouvelle espèce de *Tometes* (Teleostei: Characidae: Serrasalminae) du bouclier guyanais, *Tometes lebaili* n. sp. *Bull. Fr. Pêche. Piscic.* **364**, 23–48 (2002).

56. Agostinho, C. S., Hahn, N. S. & Marques, E. E. Patterns of food resource use by two congeneric species of piranhas (*Serrasalmus*) on the upper Paraná River floodplain. *Braz. J. Biol.* **63**, 177–182 (2003).

57. Ferreira, F. S., Vicentin, W., Costa, F. E. S. & Súarez, Y. R. Trophic ecology of two piranha species, *Pygocentrus nattereri* and *Serrasalmus marginatus* (Characiformes, Characidae), in the floodplain of the Negro River, Pantanal. *Acta Limnol. Bras.* **26**, 381–391 (2014).

58. Shirai, S. & Nakaya, K. Functional morphology of feeding apparatus of the cookie-cutter shark, *Isistius brasiliensis* (Elasmobranchii, Dalatiinae). *Zool. Sci.* **9**, 811–821 (1992).

59. Strasburg, D. W. The diet and dentition of *Isistius brasiliensis*, with remarks on tooth replacement in other sharks. *Copeia* **1**, 33–40 (1963).

60. Jones, E. C. *Isistius brasiliensis*, a squaloid shark, the probable cause of crater wounds on fishes and cetaceans. *Fish. Bull.* **69**, 791–798 (1971).

61. Papastamatiou Y. P., Wetherbee, B. M., O'Sullivan, J., Goodmanlowe, G. D. & Lowe, C. G. Foraging ecology of cookiecutter sharks (*Isistius brasiliensis*) on pelagic fishes in Hawaii, inferred from prey bite wounds. *Env. Biol. Fish.* **88**, 361–368 (2010).

62. Kosma, R. *The dentition of recent and fossil scincomorphan lizards (Lacertilia, Squamata) – Systematics, functional morphology, palecology*. PhD thesis (University of Hanover, 2004).

63. Cooper, J. S. & Poole, D. F. G. The dentition and dental tissues of the agamid lizard, *Uromastyx*. *J. Zool.* **169**, 85–100 (1973).

64. Cooper, J. S., Poole D. F. G. & Lawson R. The dentition of agamid lizards with special reference to tooth replacement. *J. Zool.* **162**, 85–98 (1970).

65. Čerňansky, A., Augé, M. L. & Rage, J.-C. A complete mandible of a new amphisbaenian reptile (Squamata, Amphisbaenia) from the late middle Eocene (Bartonian, MP16) of France. *J. Vertebr. Paleontol.* **35**, e902379 (2015).

66. Klembara, J., Hain, M. & Dobasova, K. Comparative anatomy of the lower jaw and dentition of *Pseudopus apodus* and the interrelationships of Species of subfamily Anguinae (Anguimorpha, Anguidae). *Anat. Rec.* **297**, 516–544 (2014).

67. Folie, A., Smith, R. & Smith, T. New amphisbaenian lizards from the Early Paleogene of Europe and their implications for the early evolution of modern amphisbaenians. *Geol. Belg.* **16**, 227–235 (2013).

68. Nursall, J. R. in *Mesozoic Fishes 2 – Systematics and Fossil Record* (eds Arratia, G. & Schultze, H.-P.) 189–214 (Verlag Dr. Friedrich Pfeil, 1999).

69. Friedman, M. Ray-finned fishes (Osteichthyes, Actinopterygii) from the type Maastrichtian, the Netherlands and Belgium. *Scripta Geol., Spec. Issue* **8**, 113–142 (2012).

70. Poyato-Ariza, F. J. & Bermúdez-Rochas, D. D. New pycnodont fish (*Arcodonichthys pasiegae* gen. et sp. nov.) from the Early Cretaceous of the Basque-Cantabrian Basin, northern Spain. *J. Vertebr. Paleontol.* **29**, 271–275 (2009).

71. Nursall J. R. in *Mesozoic Fishes 1 – Systematics and Paleoecology* (eds Arratia, G. & Viohl, G.) 125–152 (Verlag Dr. Friedrich Pfeil, 1996).

72. Nursall, J. R in *Origin and Phylogenetic Interrelationships of Teleosts* (eds Nelson, J. S., Schultze, H.-P. & Wilson, M. V. H.) 37–60 (Verlag Dr. Friedrich Pfeil, 2010).

73. Ørvig, T. Microstructure and growth of the dermal skeleton in fossil actinopterygian fishes: *Nephrotus* and *Colobodus*, with remarks on the dentition in other forms. *Zool. Scr.* **7**, 297–326 (1978).

74. Murray, A. M., Cook, T. D., Attia, Y. S., Chatrath, P. & Simons, E. L. A freshwater ichthyofaunal from the late Eocene Birket Qarun Formation, Fayum, Egypt. *J. Vertebr. Paleontol.* **30**, 665–680 (2010).

75. Otero, O. *et al.* A fish assemblage from the middle Eocene from Libya (Dur At-Talah) and the earliest record of modern African fish genera. *PLoS ONE* **10**: e0144358 (2015).
76. Bertaco, V. A., Malabarba, L. R. & Dergam, J. A. New *Hyphessobrycon* from the upper rio Pardo drainage in eastern Brazil (Teleostei: Characiformes: Characidae). *Neotrop. Ichthyol.* **5**, 245–249 (2007).
77. Weiss, F. E., Malabarba, M. C. & Malabarba, L. R. A new stem fossil characid (Teleostei: Ostariophysi) from the Eocene–Oligocene of southeastern Brazil. *Neotrop. Ichthyol.* **12**, 439–450 (2014).
78. Yamaoka, K. Feeding behaviour and dental morphology of algae scraping cichlids (Pisces: Teleostei) in Lake Tanganyika. *Afr. Stud. Monogr.* **4**, 77–89 (1983).
79. Gibson, S. Z. Evidence of a specialized feeding niche in a Late Triassic ray-finned fish: evolution of multidenticulate teeth and benthic scraping in †*Hemicalypterus*. *Sci. Nat.* **102**: 10 (2015).
80. Richter, M. Dental histology of a characoid fish from the Plio-Pleistocene of Acre, Brazil. *Zool. Scr.* **13**, 69–79 (1984).
81. Hibbard, C. W. A new pycnodont fish from the Upper Cretaceous of Russell County, Kansas. *Univ. Kans. Sci. Bull.* **26**, 373–375 (1939).
82. Taverne, L. Ostéologie, phylogénèse et systématique des téléostéens fossiles et actuels du super-ordre des ostéoglossomorphes. Première partie. *Acad. Roy. Belg., Mém. Cl. Sci., coll. in-8°, 2e sér.* **42**, 1–235 (1977).
83. De Schepper, N., Adriaens, D. & De Kegel, B. *Moringua edwardsi* (Moringuidae: Anguilliformes): cranial specialization for head-first burrowing? *J. Morphol.* **266**, 356–368 (2005).
84. Castle, P. H. J. & Williamson, G. R. Systematics and distribution of eels of the *Muraenesox* group (Anguilliformes, Muraenesocidae): a preliminary report and key. *J. L. B. Smith Inst. Ichthyol. Spec. Publ.* **15**, 1–9 (1975).
85. Loomis, F. B. Die Anatomie und die Verwandtschaft der Ganoid- und Knochen-Fische aus der Kreide-Formation von Kansas, U.S.A. *Palaeontographica* **46**, 213–283 (1900).
86. Hay, O. P. On certain genera and species of North American Cretaceous actinopterous fishes. *Bull. Am. Mus. Nat. Hist.* **19**, 1–95 (1903).
87. Friedman, M. *et al.* 100-million-year dynasty of giant planktivorous bony fishes in the Mesozoic seas. *Science* **327**, 990–993 (2010).
88. Bellwood, D. R. A Phylogenetic study of the parrotfishes family Scaridae (Pisces: Labroidei), with a revision of genera. *Rec. Aust. Mus.* Suppl. **20**, 1–86 (1994).

89. Newbrey, M. G., Murray, A. M., Wilson, M. V. H., Brinkman, D. B. & Neuman, A. G. Seventy-five-million-year-old tropical tetra-like fish from Canada tracks Cretaceous global warming. *Proc. R. Soc. B.* **276**, 3829–3833 (2009).

90. Bardack, D. & Sprinkle, G. Morphology and relationships of saurocephalid fishes. *Fieldiana Geol.* **16**, 297–340 (1969).

91. Cavin, L., Forey, P. L. & Giersch, S. Osteology of *Eubiodectes libanicus* (Pictet & Humbert, 1866) and some other ichthyodectiformes (Teleostei): phylogenetic implications. *J. Syst. Palaeontol.* **11**, 115–177 (2013).

92. Day, J. J. Phylogenetic relationships of the Sparidae (Teleostei: Percoidei) and implications for convergent trophic evolution. *Biol. J. Linn. Soc.* **76**, 269–301 (2002).

Supplementary Tables

Supplementary Table S1. Stratigraphic origin of the specimens of *Serrasalmimus secans* gen. et sp. nov. within the Ouled Abdoun Basin series. The stratigraphic origin of the specimens not excavated *in situ* (i.e., surface-collected material from mixed Paleogene phosphate deposits; indicated by an asterisk) has been inferred from the color of the fossil and the characteristics of the matrix.

**Specimen number**	**Stratigraphic origin**
**OCP DEK-GE 701**	Upper bone bed of Bed IIa*
M**HNM KHG 164**	Upper bone bed of Bed IIa*
M**HNM KHG 163**	Lower bone bed of Bed IIa*
M**HNM KHG 162**	Intercalary Beds II/I (possibly reworked from Bed IIa)
M**HNM KHG 161**	Upper bone bed of Bed IIa*
M**HNM KHG 160**	Upper bone bed of Bed IIa*
**OCP DEK-GE 702**	Lower bone bed of Bed IIa*
M**HNM KHG 159**	Bed I or upper bone bed of Bed IIa*
M**HNM KHG 158**	Lower bone bed of Bed IIa*
M**HNM KHG 157**	Upper bone bed of Bed IIa*
M**HNM KHG 156**	Lower bone bed of Bed IIa*
M**HNM KHG 155**	Lower bone bed of Bed IIa*
M**HNM KHG 152**	Bed I or upper bone bed of Bed IIa*
M**HNM KHG 113**	Lower bone bed of Bed IIa

Supplementary Table S2. Data matrix used for the phylogenetic analysis.

Outgroup
0000000000 0000000000 0000000000 0000000000 0000000?00 0000000?00 00?0000000 0000000000 0000000000 1000000000 00?0?10200 1

Akromystax
0001021010 1010000??? 2402?43?51 4?0251??01 0221220221 0200240413 2252300010 3021224021 4205530001 1?51121102 00?1?0000? 0

Coelodus
120?001?0? ??1???0??? 2????3??21 230?4133?0 1111220?22 120?250114 ?05330?0?? 320000???? ??0??????0 1?????1?02 00?100000? ?

Iemanja
1?0?010000 0010??0??? ?311???532 2?0?20??10 0214310213 020?13??13 30?33010?0 ?0??????22 220????0?? 1??1221?02 00?1000000 0

Neoproscinetes
0001101000 00100?0121 2311232321 4213113120 0112221213 020023?112 30333010?0 3213122122 120523?000 1121221?02 00?1000000 0

Ocloedus
0202100000 1010010121 2401232?21 2304113211 1201220211 0201200112 2253300010 3122223032 2205330000 1?21?21102 0001000000 0

Oropycnodus
0201000000 101001112? 24?2?32?21 ?104113111 0222230122 0200350414 305430(012)021 4100004122 2205232100 1??1?21102 0001?00000 0

Polazzodus
1101001000 0010001??? 2402?34641 2104113{12}11 1211230132 020(01)240113 ?03430(02)010 3100003022 1205331000 1?0???1?02 ?0?100000? 0

Potiguara
020210000? 1010??0??? ???????21 230?113?11 ?1?1{12}20212 0?0?25???4 2????????2? ??0000??21 220??????0 1??1????0? ????00000? 0

Proscinetes
020110(01)000 0010010121 2301132321 4213113210 0111220212 0200241103 203330(12)020 2200002120 00052300?0 0111221?02 0001000000 0

Pycnodus
1000001000 1010001130 2401232311 2204113310 0222232122 0200440003 405430(02)021 4100004132 1205341000 1?11221102 0001?00000 0

Rhinopycnodus
1001100000 0010010??1 2301??43?? ???4????1? ?12???0102 020014??03 3353300010 212111??11 120?530000 1?21?21102 00?1?0000? ?

Stemmatodus
1000100000 00100?0121 2401232411 2204113311 0201230202 0200001012 2234302010 3032224021 2205350000 1?11221102 0001000000 0

Stenamara
3041100000 00100?012? ??01?????? ????11421? 0201220303 0202001?00 22?330?010 30212220?? ?204340000 1?21????02 000100000? ?

Tepexichthys
0201100000 1010010122 2301?31311 1303113220 0101221212 0200200103 3033301010 2000002021 2203240000 11?1??1102 0001000000 0

Turbomesodon
0200100000 0010010121 2301?32321 ?304113111 1201220302 02001(03)1212 2253302010 2(12)32122022 2203340010 1131????02 00?100000? ?

Abdobalistum
000?001??0 0?10??013? ???1?2???1 ????113??1 1012231?22 020?250004 442421?022 2011103000 001??????0 1?41?21?02 ?001???0?? 0

Nursallia
02000011?0 0010??0??? ???1?22?11 4204?1?2?0 0223??2223 02002501?4 436120(01)023 31000?41?? ??04??10?0 1?41??1102 00?1???0?? 0

Paranursallia
0200001110 00?0000030 2301?22111 2104113210 0?23232223 02002501?4 ??63201020 21????30?? 1205???0?0 1?41?2110? ?0???0000? 0

Palaeobalistum
1100001??0 ?0?0010?10 ???1????11 ?20?1132?0 00??????12 0?002???1? ?3?00100?0 2111102031 1?0??????0 1?4?????02 10?1????0? ?

Coccodus
2100001000 0001000??? ?????32?41 4204114231 13112?0112 0411?105?5 22?543{01}?34 6354336243 3327460002 ???1??1?02 ?00200000? ?

Ichthyoceros
0001011012 00020?01?0 1??123??41 4304313301 1????????? ?33121?035 0120023220 50110?5011 ?206340000 ???1?2??02 0001?0000? ?

Arduafrons
0011011?00 0?00000110 11?1?12121 ?3021??300 00000????? ?100130022 0340000010 1031111031 12011100?? ???1?11?02 1111?0?00? 0

Brembodus
0021001100 00000?0110 1101121?21 2202111210 0221100203 0201401{14}20 1220000010 30311120?? ??051100?? ???1?11100 0111?00000 0

Gibbodon
0011001000 0000010000 21?1021?21 4301?????1 0????????? ?201210025 02?0000010 3021112031 1?031100?? ???1?00100 0111?0?00? 0

Eomesodon
00310?1100 0000010?00 ?1?122???? ???41???11 0200??0?00 0?0??????1 ???03000?0 ?03111?000 ??0??????? ???1????02 00?1?0000? ?

Apomesodon
0020110000 0000000?{12}? ?1?1222?11 ?30?1132?1 00001?0301 0200200421 124030{03}010 1031111021 12021100?? ???1?21?01 0011?0000? ?

Gyrodus
0101001100 0000000010 1101212111 2302112212 1101410112 0202200124 3010000010 2031111021 11011100?? ???1111212 010100001? 0

Polygyrodus
?????????? ?????????? ????????1{01} 220?2{01}12?2 0????????? ?????????? ?????????? ?????????? ?????????? ????????1? ????110010 0

Damergouia
?????????? ?????????? ????????11 2?0?5{01}31?3 0????????? ?????????? ?????????? ?????????? ?????????? ????????1? ????110120 0

Eoserrasalmimus
?????????? ?????????? ????????51 320??????3 0????????? ?????????? ?????????? ?????????? ?????????? ????????1? ?????10121 1

Serrasalmimus
?????????? ?????????? ????????61 520?6153?3 0????????? ?????????? ?????????? ?????????? ?????????? ????????1? ????211221 2

Supplementary Figures

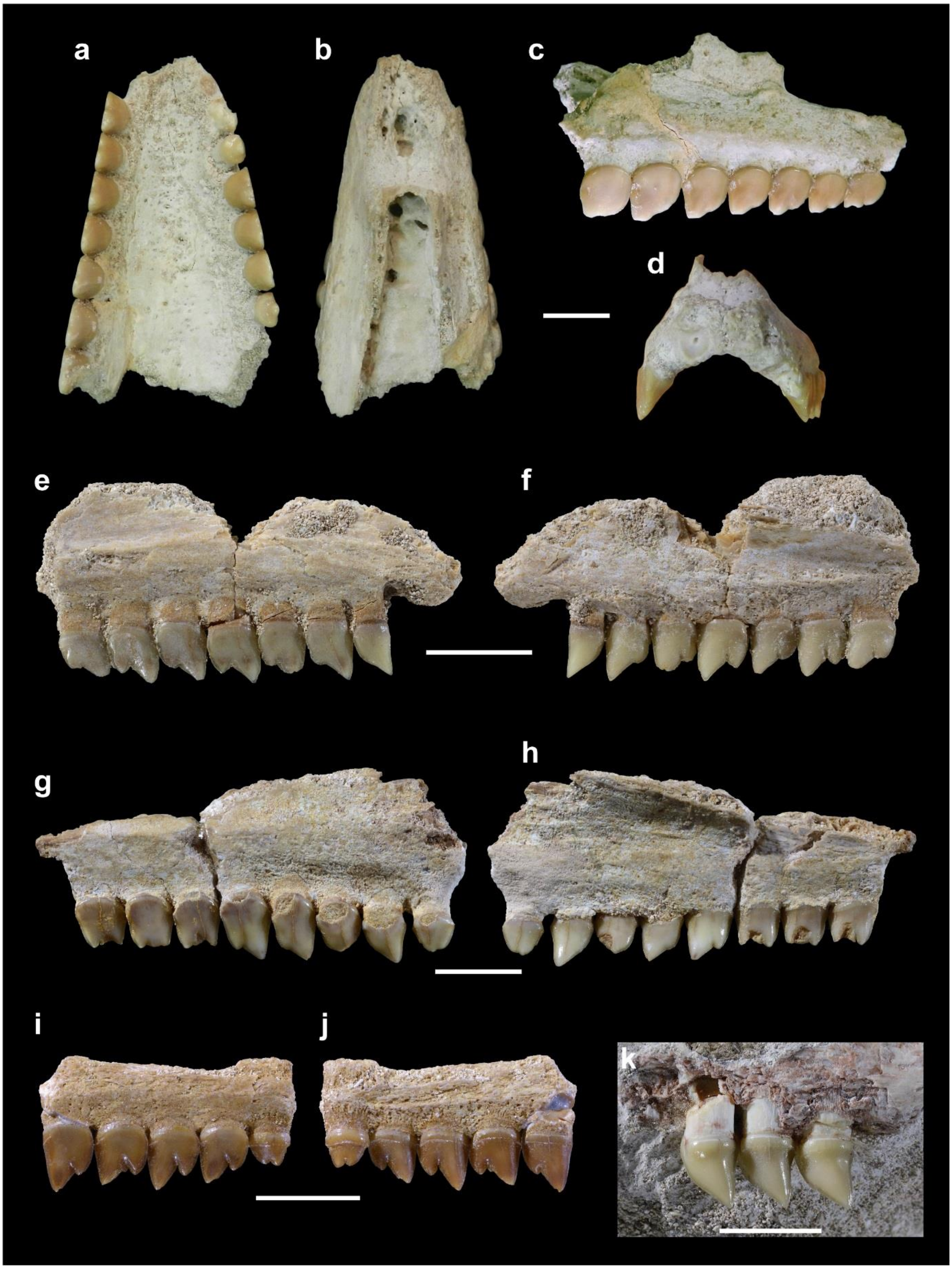

Supplementary Figure S1. Vomers of *Serrasalmimus secans* gen. et sp. nov. from the Paleogene of the Ouled Abdoun Basin, Morocco. a–d, Nearly complete vomer (OCP DEK-

GE 701) in ventral (**a**), dorsal (**b**), right lateral (**c**) and anterior (**d**) views. **e**, **f**, Fragmentary vomer (MHNM KHG 160) in left lateral (**e**) and medial (**f**) views. **g**, **h**, Fragmentary vomer (MHNM KHG 164a) in left lateral (**g**) and medial (**h**) views. **i**, **j**, Fragmentary vomer (MHNM KHG 163) in right lateral (**i**) and medial (**j**) views. **k**, Fragmentary vomer (MHNM KHG 162) in right medial view; note the tubular root-like structures penetrating the bone and firmly fused to it (ankylothecodont-like tooth attachment). Scale bars, 10 mm. (**e**–**k**, photographs by Lilian Cazes – CNRS/MNHN).

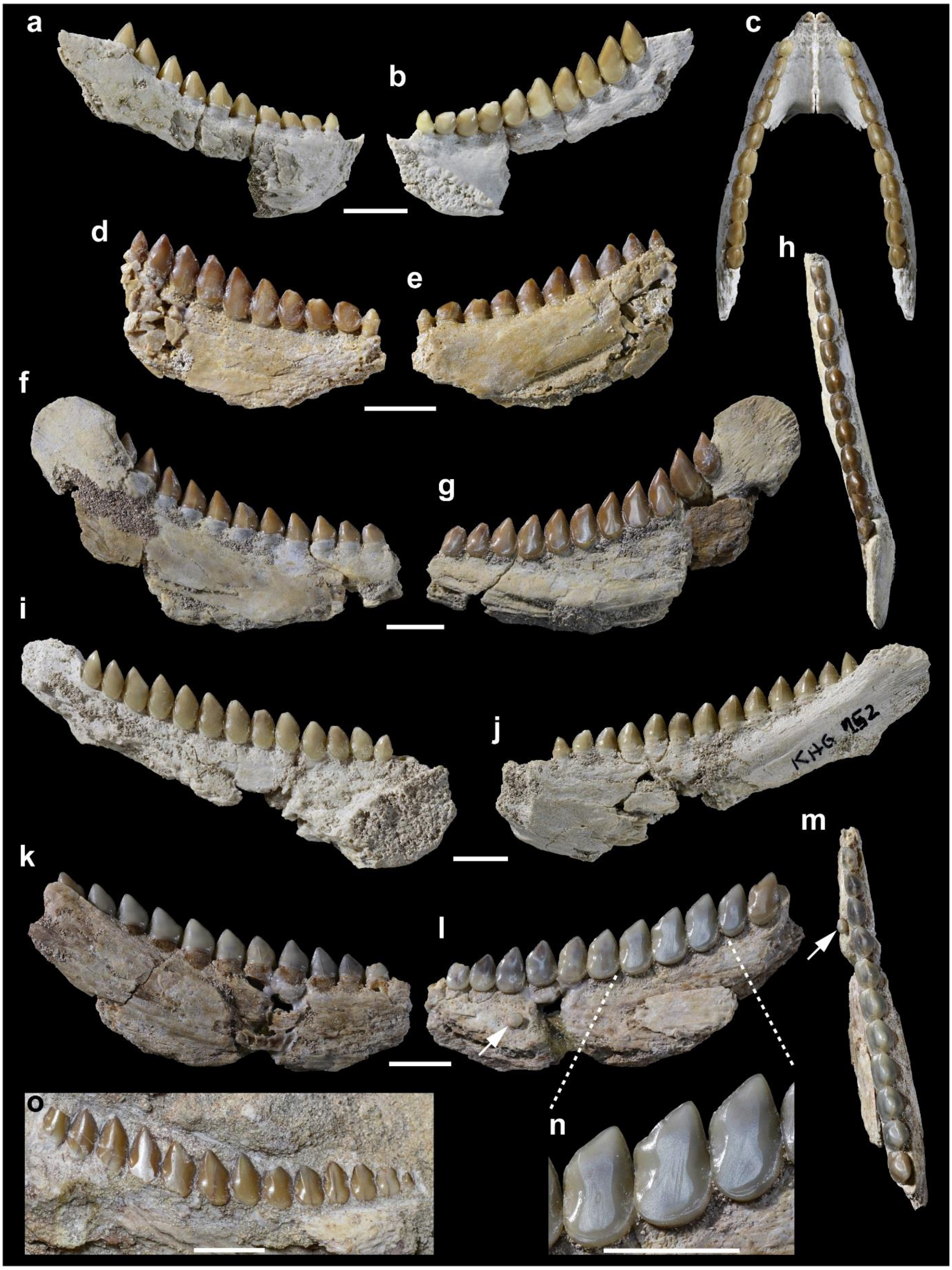

Supplementary Figure S2. Prearticulars of *Serrasalmimus secans* gen. et sp. nov. from the Paleogene of the Ouled Abdoun Basin, Morocco. a–**c**, Nearly complete right prearticular (MHNM KHG 159) in lateral (**a**), medial (**b**) and dorsal (**c**, with mirror image for the left side) views. **d**, **e**, Nearly complete left prearticular (MHNM KHG 155) in medial (**d**) and lateral (**e**)

views. **f**–**h**, Nearly complete right prearticular (MHNM KHG 158) in lateral (**f**), medial (**g**) and dorsal (**h**) views. **i**, **j**, Nearly complete left prearticular (MHNM KHG 152) in medial (**i**) and lateral (**j**) views. **k**–**n**, Nearly complete right prearticular (MHNM KHG 157) in lateral (**k**), medial (**l**) and dorsal (**m**) views, and close-up of tooth crowns showing wear facets and striae of the lingual surface (**n**); note the vestigial tooth, remnant of one of the medial tooth rows lost during serrasalmimid evolution (arrow in **l** and **m**). **o**, Nearly complete left prearticular (MHNM KHG 156) in medial view. Scale bars, 10 mm. (Photographs by Lilian Cazes – CNRS/MNHN).

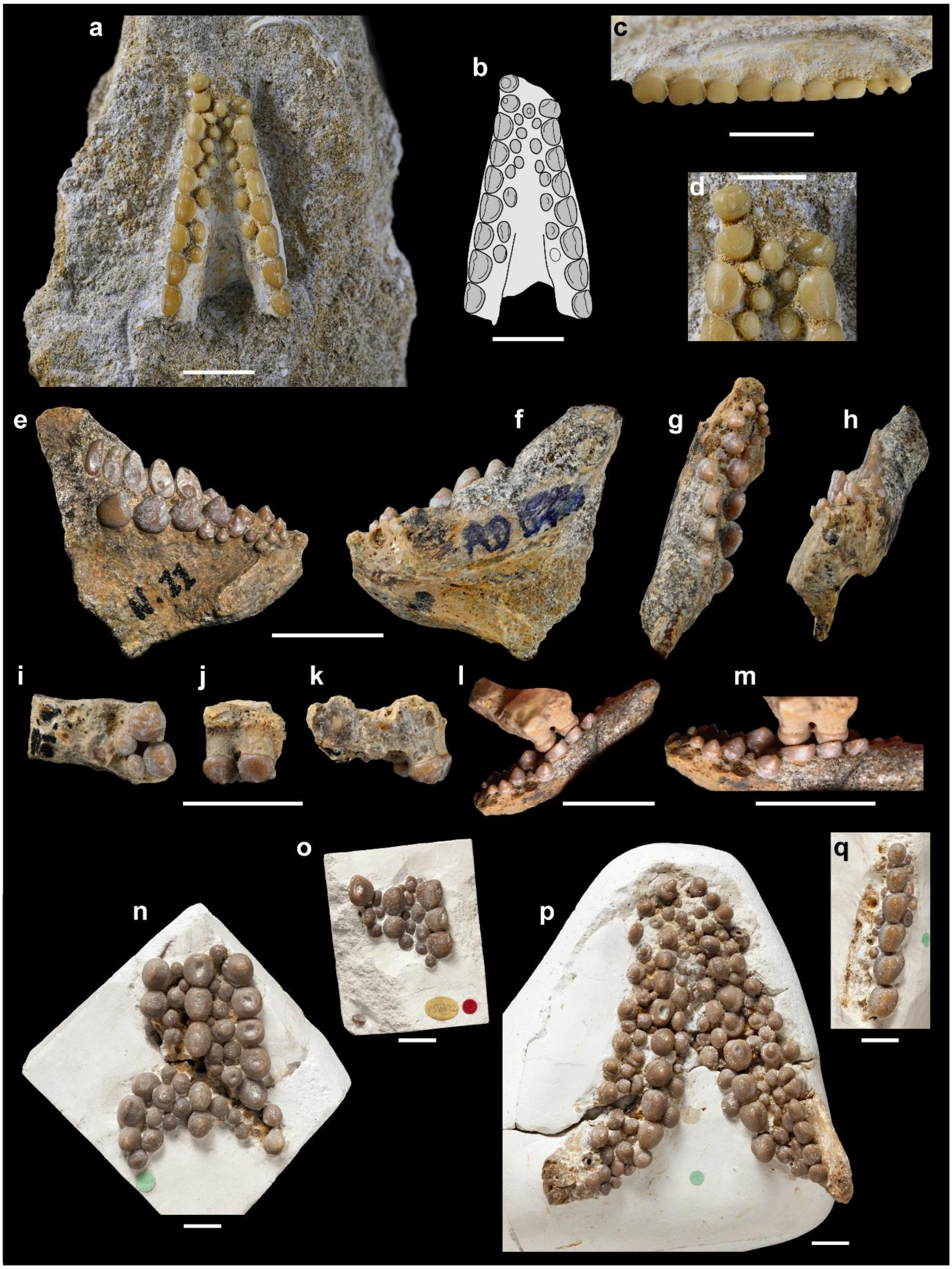

Supplementary Figure S3. Jaw elements of the Cretaceous Serrasalmimids. a–d, *Eoserrasalmimus cattoi* gen. et sp. nov. from the Late Cretaceous (Maastrichtian) of the Ouled Abdoun Basin, Morocco. Complete vomer (MHNM KHG 165) in ventral (**a**, **b**, photograph and interpretative drawing, respectively) and right lateral (**c**) views, and detail of tooth arrangement

and morphology in the anterior portion (**d**). **e**–**m**, *Damergouia lamberti* gen. et sp. nov. from the Late Cretaceous (Turonian) of the Damergou area, Niger. Complete left prearticular (MNHN.F.HGS176) (**e**–**h**) in medial (**e**), lateral (**f**), dorsal (**g**) and anterior (**h**) views. Fragmentary vomer (MNHN.F.HGS177) (**i**–**k**) in ventral (**i**), left lateral (**j**) and anterior (**k**) views. Vomerine and prearticular dentitions placed in occlusion (**l**, **m**), in anterior oblique (**l**) and left lateral (**m**) views. **n**–**q**, *Polygyrodus cretaceus* (Agassiz, 1843) from the Late Cretaceous of England. Nearly complete vomerine dentition (NHMUK PV OR 39048) (**n**) from the Turonian (Middle Chalk) of Lewes, Sussex. Fragmentary vomerine dentition (NHMUK PV OR 49802) (**o**) from the Coniacian–?Campanian (Upper Chalk) of Guildford, Surrey. Complete, associated right and left prearticular (and dentary?) dentitions (NHMUK PV P 11157) (**p**) from the Turonian (Middle Chalk) of Cuxton, Kent. Fragmentary left prearticular dentition (NHMUK PV P 6852) (**q**) from the Cenomanian (Lower Chalk) of Warlingham, Surrey. Scale bars, 10 mm (**a**–**c**, **e**–**q**); 5 mm (**d**). (**a**, **c**–**k**, photographs by Lilian Cazes – CNRS/MNHN; **n**–**q**, copyright: The Trustees of the Natural History Museum, London – http://data.nhm.ac.uk).

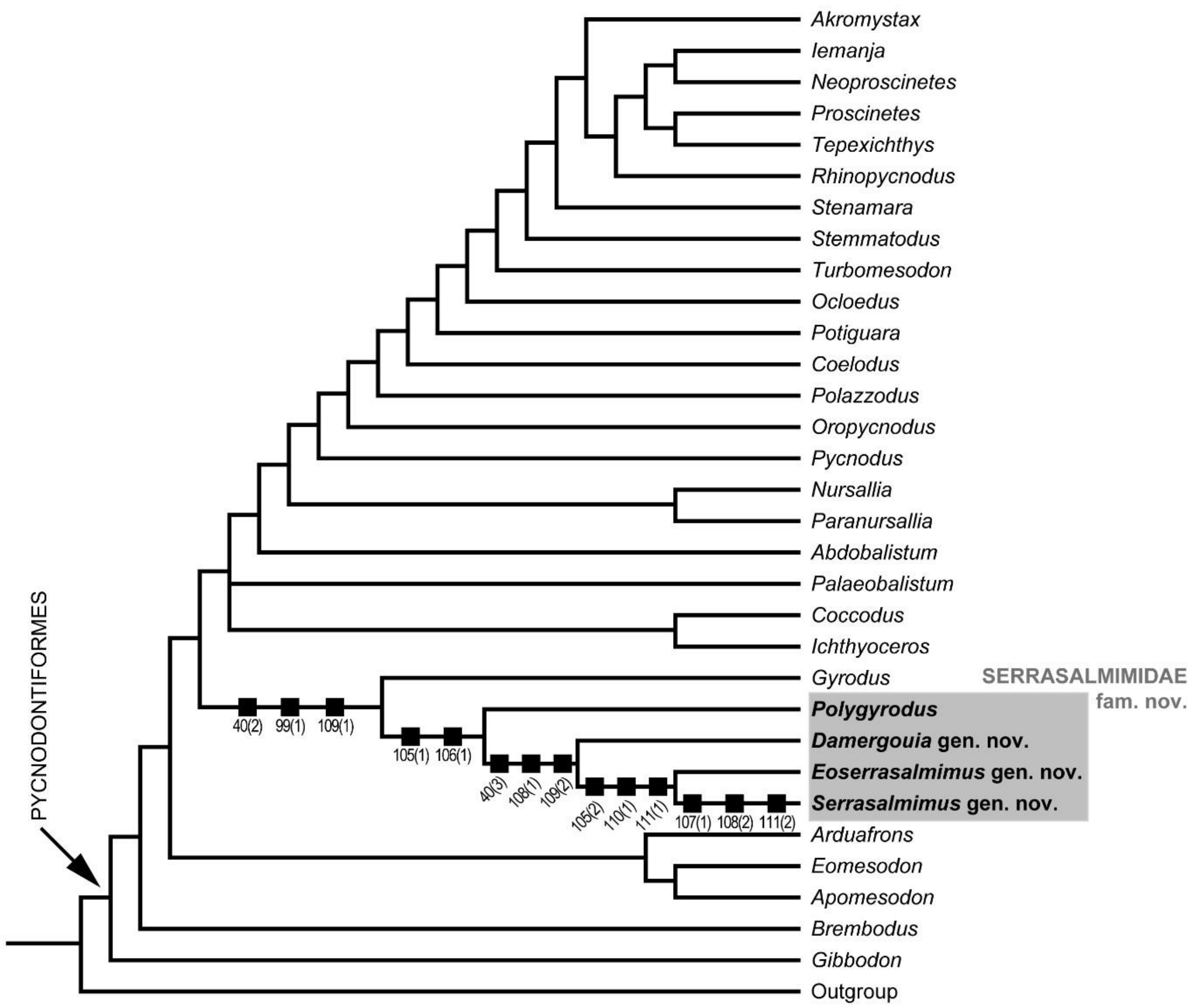

Supplementary Figure S4. Cladogram showing the tree resulting from the phylogenetic analysis (length 569, CI = 0.5114, RI = 0.4991, RC = 0.2553). Only the uniquely derived character changes affecting the new taxa are shown here (black squares, with corresponding character numbers and character states indicated below). The monophyly of the Serrasalmimidae is confirmed, and this new family is recovered as the sister group to *Gyrodus* (Gyrodontidae).

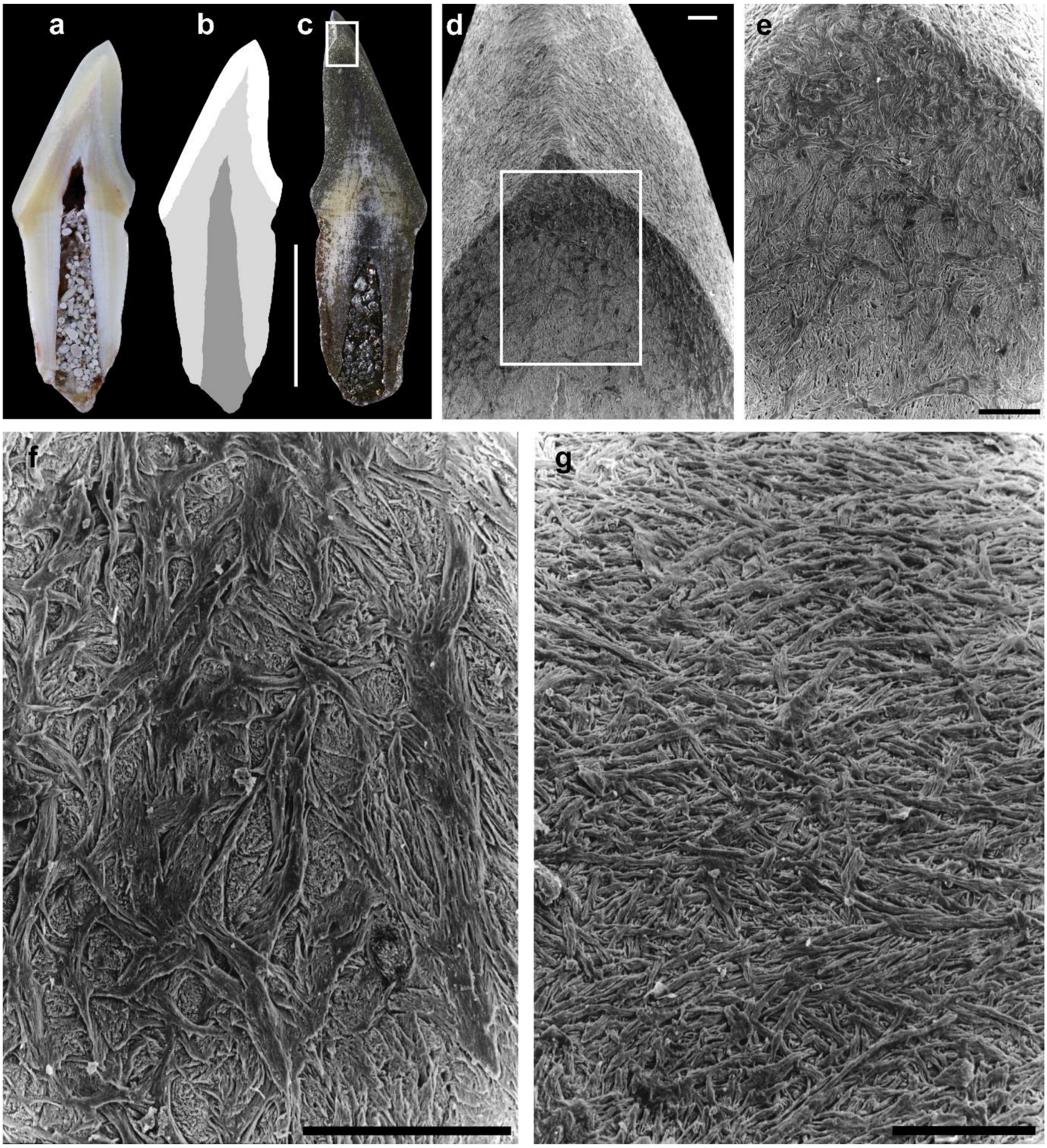

Supplementary Figure S5. Dental histology and microstructure of *Serrasalmimus secans* gen. et sp. nov. a, **b**, Vertical (longitudinal) section of the anteriormost tooth (anterior half) of the fragmentary vomerine dentition MHNM KHG 162 (**a**) and interpretative drawing showing the acrodin layer (in white), the dentine layer (in light grey) and the pulp cavity (in dark grey) (**b**). **c**, Vertical (longitudinal) section of the anteriormost tooth (posterior half) of the fragmentary vomerine dentition MHNM KHG 162 prepared (metallized) for SEM observation. **d**, SEM image of box area in c showing the thickness of the acrodin layer and the arrangement

of fibre bundles. **e**, SEM image of box area in d showing woven fibre bundles becoming densely arranged near the crown surface. **f**, SEM image showing the detail of woven fibre bundles. **g**, SEM image showing the strongly woven fibre bundles present on the crown surface. Scale bars, 5 mm (**a**–**c**); 100 µm (**d**–**g**). (**a**, **c**, photographs by Lilian Cazes – CNRS/MNHN).

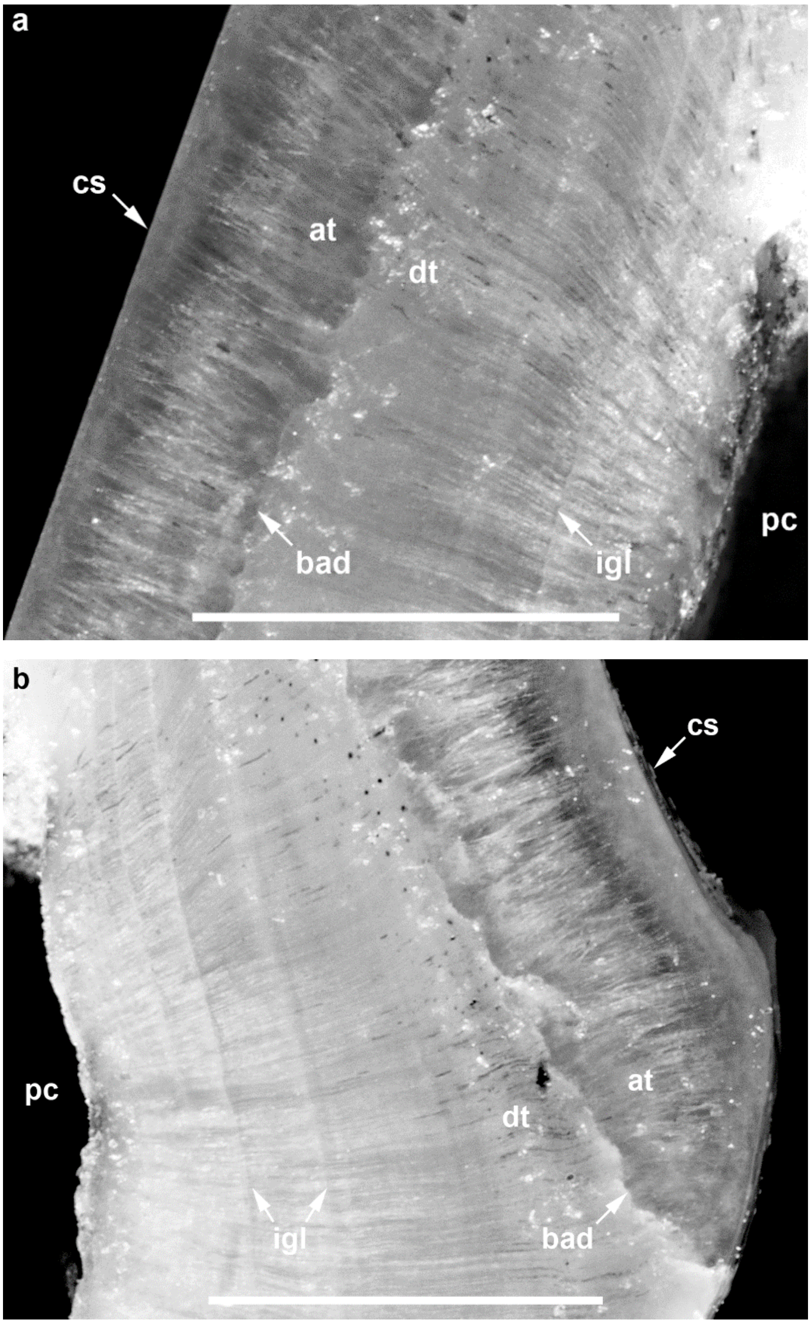

Supplementary Figure S6. Dental histology and microstructure of *Serrasalmimus secans* gen. et sp. nov. Vertical (longitudinal) section (**a**, labial side; **b**, lingual side) of the anteriormost tooth of the fragmentary vomerine dentition MHNM KHG 162 observed under special lighting conditions. Dentinal and acrodin tubules, which are sub-perpendicular to the crown surface, are revealed by a light beam oriented laterally. The thin and straight acrodin tubules mostly lie in direct continuity with the dentinal tubules and correspond to the "acrodin canals of type A" described by Ørvig 1978 ([73], fig. 63). Note that the dentine layer shows several incremental growth lines. Abbreviations: at, acrodin tubules; bad, boundary between acrodin and dentine; cs, crown surface; dt, dentinal tubules; igl, incremental growth line; pc, pulp cavity. Scale bars, 1 mm. (Photographs by Lilian Cazes – CNRS/MNHN).

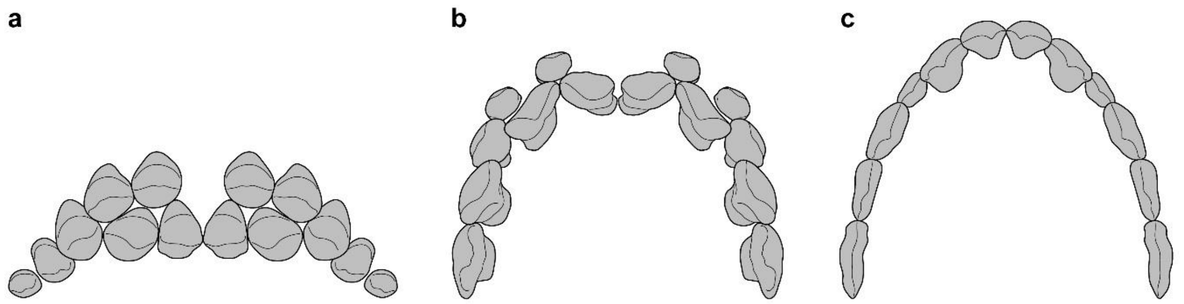

Supplementary Figure S7. Upper dentitions of the three main clades recognized within the Serrasalmidae. a, Premaxillary dentition of *Colossoma macropomum*, a member of the pacu clade, in ventral view (redrawn from ref. [13]). **b**, Premaxillary dentition of *Tometes lebaili*, a member of the *Myleus* clade, in ventral view (redrawn from ref. [55]). **c**, Premaxillary dentition of *Serrasalmus rhombeus*, a member of the piranha clade, in ventral view (redrawn from ref. [13]). Not to scale.

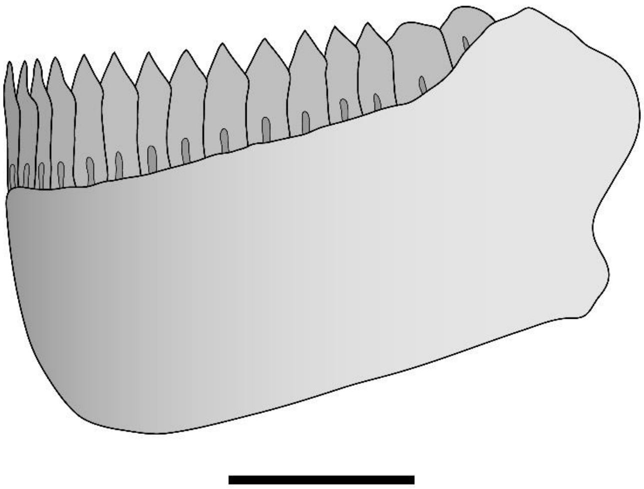

Supplementary Figure S8. Cutting dentition of the lower jaw of the cookiecutter shark *Isistius brasiliensis* (Dalatiidae). Mandible in left lateral view, showing the row of labiolingually compressed triangular teeth (redrawn from ref. [58]). Scale bar, 10 mm.

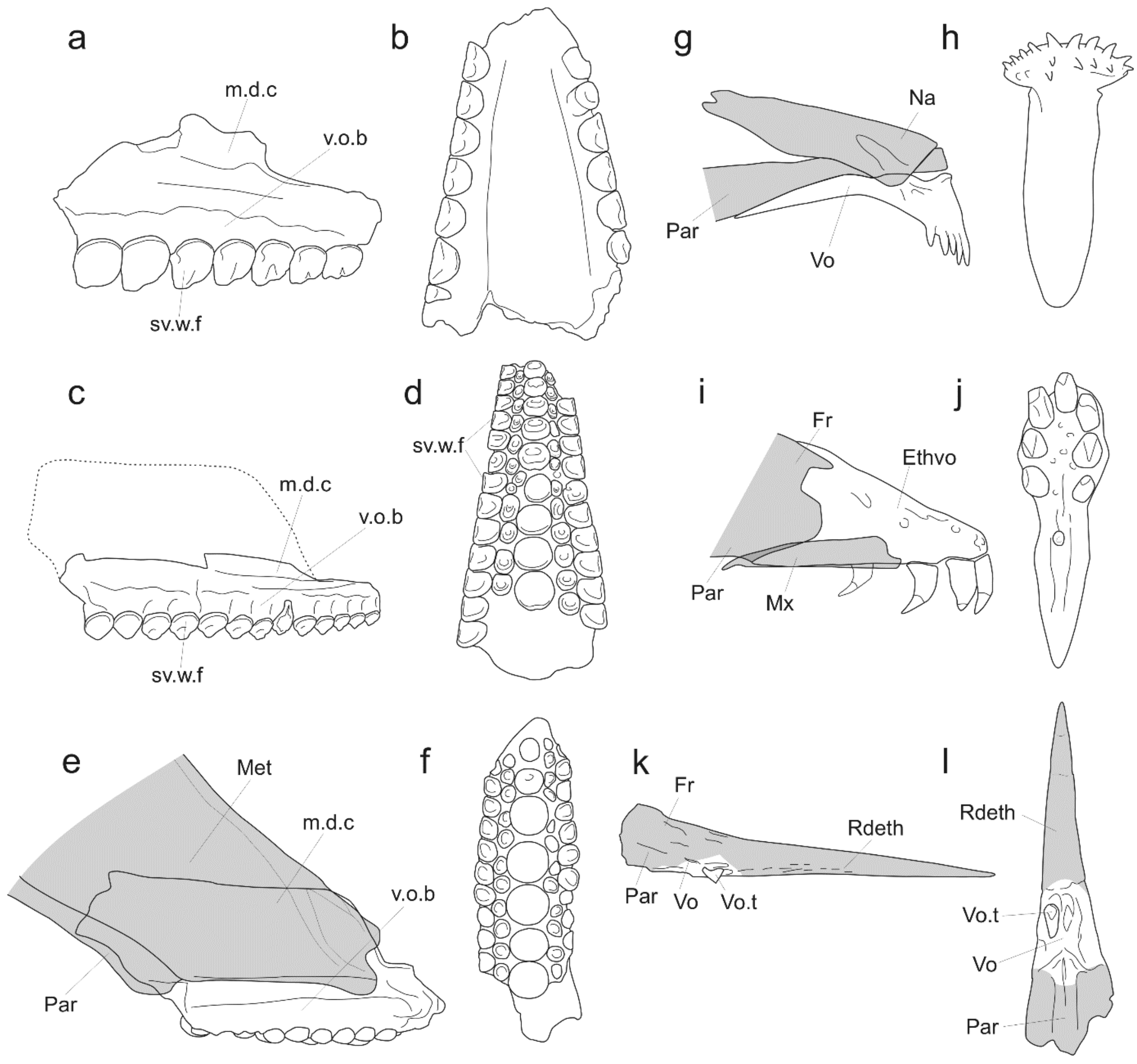

Supplementary Figure S9. Comparison between the vomer of *Serrasalmimus secans* gen. et sp. nov. and those of other ray-finned fishes. a, **b**, Vomer (OCP DEK-GE 701, holotype) of *Serrasalmimus seccans* in right lateral (**a**) and ventral (**b**) views. **c**, **d**, Vomer (IGR 24999, Géosciences Rennes) of *Gyrodus* sp., Late Jurassic (Tithonian) of Mazeray (Charente-Maritime, France) in left lateral (**c**) and ventral (**d**) views (both drawings are mirror images for homogeneity); note that in this specimen the median dorsal crest (m.d.c) is broken at its base. **e**, **f**, Vomer (unnumbered specimen, JURASSICA Museum, Porrentruy) of cf. *Gyrodus* sp., Late Jurassic of Plagne (Bern, Switzerland) in left lateral (**e**) and ventral (**f**) views, with arrangement of the mesethmoid and parasphenoid (in grey) according to ref. [68]. Shared characters between *Serrasalmimus* and *Gyrodus* are the median dorsal crest of the vomer (m.d.c), which is embraced by the mesethmoid (Met), the vertical oral border above the ventral margin (v.o.b) and the subvertical wear facet (sv.w.f). The condition of the vomer is very

different in other ray-finned fishes with an unpaired toothed ossification in the upper jaw, such as the osteoglossomorph *Scleropages formosus* (**g**, **h**, in right lateral and ventral views, respectively; redrawn from ref. [82]), the anguilliform *Moringua edwardsi* (**i**, **j**, in right lateral and ventral views, respectively; redrawn from ref. [83]), and the pachycormiform *Protosphyraena* spp. (**k**, *P. tenuis* in right lateral view; **l**, *P. nitida* in ventral views; redrawn from ref. [85] and ref. [86], respectively). Abbreviations: Ethvo, ethmovomer; Fr, frontal; m.d.c, median dorsal crest; Met, mesethmoid; Mx, maxilla; Na, nasal; Par, parasphenoid; Rdeth, rostrodermethmoid; sv.w.f, subvertical wear facet; v.o.b, vertical oral border; Vo, vomer; Vo.t, vomerine tooth. Not to scale.
